# Myxozoa in high Arctic: Survey on the central part of Svalbard archipelago

**DOI:** 10.1016/j.ijppaw.2014.02.001

**Published:** 2014-02-26

**Authors:** Alena Kodádková, Iva Dyková, Tomáš Tyml, Oleg Ditrich, Ivan Fiala

**Affiliations:** a Faculty of Science, University of South Bohemia in České Budějovice, Branišovská 31, 370 05 České Budějovice, Czech Republic; b Institute of Parasitology, Biology Centre of the Academy of Sciences of the Czech Republic, Branišovská 31, 370 05 České Budějovice, Czech Republic; c Department of Botany and Zoology, Faculty of Science, Masaryk University, Kotlářská 2, 611 37 Brno, Czech Republic

**Keywords:** Myxosporea, Marine urinary clade, Arctic, Phylogeny, *Zschokkella*, *Schulmania*

## Abstract

Myxosporeans (Myxozoa), microscopic metazoan parasitic organisms, are poorly studied in the Arctic region. Our survey of benthic and pelagic fish (*n* = 234) collected in Isfjorden (Svalbard, Norway) together with detailed morphological and molecular examination revealed the presence of nine myxosporean species. We compared observed myxosporean diversity with diversity documented in regions close to the Arctic and revealed that water depth rather than geographic distribution is an important factor influencing myxosporean fauna.

We describe three new myxosporean species: *Zschokkella siegfriedi* n. sp. from kidney of *Boreogadus saida*, *Parvicapsula petuniae* n. sp. from the urinary bladder of *Gymnocanthus tricuspis*, and *Sinuolinea arctica* n. sp. from the urinary bladder of *Myoxocephalus scorpius*. We characterise *Latyspora*-like organism from kidney of *Clupea harengus*. We provide new data for *Ceratomyxa porrecta*, *Myxidium gadi*, *Myxidium finnmarchicum*, *Schulmania aenigmatosa*, and *Parvicapsula irregularis* comb. nov. The phylogenetic analyses including the newly obtained SSU and LSU rDNA data revealed that most of the species studied cluster in the marine urinary clade within the marine myxosporean lineage. Newly obtained sequences including the first molecular data for the member of the genus *Schulmania*, substantially enriched the *Zschokkella* subclade. *C. porrecta* and the two *Myxidium* species cluster within the *Ceratomyxa* and marine *Myxidium* clade, respectively.

Newly described species, *Z. siegfriedi* n. sp., was revealed to be morphologically indistinguishable but genetically diverse from *Zschokkella hildae* known from numerous gadid fish. Therefore, we consider *Z. siegfriedi* to be a cryptic myxosporean species that might be misidentified with *Z. hildae*. A *Latyspora*-like organism was found to be taxonomically problematic due to its suture line and its distant phylogenetic position from the type species *Latyspora scomberomori* did not allow us to assign it to the genus *Latyspora*. Based on an increased taxon sampling and SSU + LSU rDNA-based phylogeny, evolutionary trends within the marine urinary clade are investigated.

## Highlights

• Morphological and molecular characterisation for nine myxosporeans is provided.
• Comparison of myxosporean diversity from the Arctic with other regions is performed.
• The taxon sampling of the marine urinary clade is markedly increased.
• Evolutionary trends within the marine urinary clade are discussed.

## Graphical abstract

**Figure:**
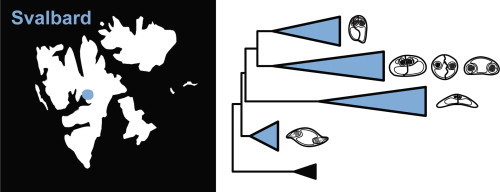

## 1 Introduction

Arctic ecosystems draw our attention due to their rapid responses to climate change (Post et al., 2009). The Arctic region can be defined as north of the Arctic Circle, and consists the Arctic Ocean, northern part of Eurasia and North America, Greenland, Iceland, Svalbard archipelago etc. The Arctic can be divided into the Low Arctic and High Arctic, according to various environmental and biological characteristics. The Svalbard archipelago is located in the High Arctic. The Arctic Ocean is the most extreme ocean in regard to the seasonality of light and its seasonally fluctuating ice cover. In general, species richness is lower in the Arctic than at lower latitudes and is to some degree constrained by biotic and abiotic mechanisms that define species occurrences and associations (Hoberg and Kutz, 2013). Furthermore, species richness tends to decline from low to high Arctic (Payer et al., 2013). Low numbers of host species is usually correlated to low numbers of parasites. Moreover, water temperature may influence transmission dynamics and parasite development (e.g. Kerans et al., 2005). Arctic fjords in the west coast of the Svalbard archipelago region are exceptional in terms of significantly higher temperatures caused by the Gulf Stream. Variations in the number of parasites were found in morphotypes of threespine sticklebacks living in different temperatures; higher numbers of parasites were found in the morphotype from the deep-cold water habitat compared to two warmer water dwelling morphotypes in the same Iceland lake (Karvonen et al., 2013). The enriching effect of warmer temperatures on higher abundance and species richness of ectoparasites was demonstrated in more than 100 fish hosts. This effect is not an artefact, but rather an indication of the importance of temperature in the diversification of fish parasites in the tropics (Poulin and Rohde, 1997).

Myxosporean fauna has been poorly studied in the Arctic region. One of the most parasitologically and ecologically studied marine fish with high economical importance occurring in sub-Arctic and Arctic waters is the Atlantic cod *Gadus morhua* (Hemmingsen and MacKenzie, 2001, Perdiguero-Alonso and D., 2008)*.* Apart from a number of protozoan and metazoan parasites (mostly helminths), 11 myxosporean species have been found in Atlantic cod (Hemmingsen and MacKenzie, 2001, Køie et al., 2007a, Holzer et al., 2010). A survey of parasite fauna of Atlantic cod revealed relatively rich and abundant regional macroparasite fauna dominated mostly by generalist parasites with Arctic-Boreal distribution in six localities in the North East Atlantic (Perdiguero-Alonso et al., 2008). These high-level fauna comparisons suggest that differences in the feeding behaviour of cod amongst localities which could affect the prevalence and abundance of parasite species. Kerans et al. (2005) found that water temperature influenced parasite development rates and was a primary determinant for the release of actinospores of the myxozoan *Myxobolus cerebralis* in strains of its definitive host *Tubifex tubifex*. In addition to latitudinal temperature gradients, sea depth is an important factor for parasite ecology. Low parasite richness was reported in different meso- and bathypelagic fishes in comparison to benthopelagic species in the Arctic Ocean (Klimpel et al., 2006).

This study is focused on the Myxozoa, microscopic metazoan parasites characterised by simplified bodies. Evolutionary history of the Myxozoa has been questioned until recent molecular evidence proved the cnidarian origin (Jiménez-Guri et al., 2007, Holland et al., 2011). Myxozoans infect various organs in the vertebrate, mainly fish, hosts: coelozoic species multiply in the cavities of body organs (gall bladder, urinary tract, renal corpuscles etc.) whereas histozoic species are intercellular in various tissues (liver, skin, kidney, testes etc.). The phylum Myxozoa is divided into two classes: Malacosporea with only three described species and Myxosporea with the overwhelming majority of the myxozoan species. Until now, approximately 2310 myxosporean species assigned to 60 genera have been described (Morris, 2010). Myxosporean genera are characterised by the morphology of the spore: spore shape, number of spore valves and polar capsules (PCs), and position of suture lines towards the PCs are considered the main taxonomic features. However, many myxospore morphological features are not synapomorphic since great discrepancies were found between the classic taxonomic approach and the phylogenetic relationships (Holzer et al., 2004, Fiala and Bartošová, 2010).

Myxosporeans form two main phylogenetic lineages according to host habitat, i.e. marine and freshwater (Fiala, 2006), plus a recently revised third basal sphaerosporid lineage (Bartošová et al., 2013). The marine lineage exclusively consists of marine species with the exception of *Ceratomyxa shasta*. There are five clades within the marine lineage: the marine *Myxidium* clade, the *Ceratomyxa* clade, the *Enteromyxum* clade*,* the *Kudoa* clade and the marine urinary clade divided into the *Parvicapsula* and *Zschokkella* subclade (Fiala, 2006, Bartošová et al., 2011). With the exception of the *Enteromyxum* clade, the remaining clades include non-monophyletic genera. The clustering of species in particular clades follows tissue tropism criterion rather than myxospore morphology (Holzer et al., 2004, Fiala, 2006). The marine urinary clade is typical in this respect: phylogenetically closely related myxosporeans of the genera *Parvicapsula*, *Gadimyxa*, *Sphaerospora*, *Sinuolinea*, *Latyspora*, and *Zschokkella* differ in spore morphology but predominately infect the excretory tract (Bartošová et al., 2011). However, some species of the *Parvicapsula* subclade also infect other sites such as the epithelium of the gall bladder, the intestine, the pseudobranchs and testicles. The monophyly of the genus *Parvicapsula* was disrupted by clustering of *Gadimyxa* spp. with parvicapsulids as well as by the sister relationship of *P. minibocornis* and *Sphaerospora testicularis* (Køie et al., 2007a, Bartošová et al., 2011). The *Zschokkella* subclade contains species of the polyphyletic genus *Zschokkella* including its type species *Z. hildae* as well as type species of the genera *Latyspora* and *Sinuolinea* (Bartošová et al., 2011, Dyková et al., 2013). The *Zschokkella* subclade is characterised by species with high variability in myxospore shape with the position of PCs ranging from set at opposite ends of the spore, to directly next to each other.

This paper attempts to characterise myxosporean fauna on the Svalbard archipelago: (i) detailed morphological and molecular characterization of myxosporean species; (ii) phylogeny and evolutionary trends; (iii) comparison of parasite diversity from the Arctic with other regions.

## 2 Material and methods

### 2.1 Fish hosts

Eight species of teleost fish were collected in part of the Billefjorden, Isfjorden, Petunia Bay (78° 69′ N, 16° 53′ E) in the central part of Svalbard archipelago during the summer season (July and August 2011). A total of 234 individuals of 8 fish species from 7 families were dissected. Families, namely Cottidae: *Myoxocephalus scorpius* (Linnaeus, 1758) (*n* = 98), *Gymnocanthus tricuspis* (Reinhardt, 1830) (*n* = 22); Clupeidae *Clupea harengus* Linnaeus, 1758 (*n* = 66); Osmeridae: *Mallotus villosus* (Müller, 1776) (*n* = 16); Gadidae: *Boreogadus saida* (Lepechin, 1774) (*n* = 14); Pleuronectidae: *Hippoglossoides platessoides* (Fabricius, 1780) (*n* = 9); Myctophidae: *Lumpenus lampretaeformis* (Walbaum, 1792) (*n* = 8); and Salmonidae: *Salmo salar* Linnaeus, 1758 (*n* = 1). Fish were caught using gillnets in littoral habitat (maximum depth of gillnets was 40 m). After euthanasia all organs were checked for the presence of the Myxozoa in squash preparations by light microscopy (Olympus BX 53). Contents of gall and urinary bladders were examined fresh, under cover slips, on slides covered with a thin layer of 1% agar. In some cases we failed to obtain samples of gall and urinary bladders and the missing data are considered in prevalence records within species descriptions. A DNA sample of *Parvicapsula minibicornis* was obtained from the kidney of *Oncorhynchus nerka* (Walbaum, 1792) in Cultus Lake (British Columbia, Canada).

### 2.2 Myxosporean collection and documentation

Pictures of fresh spores were made using an Olympus BX 53 microscope with Nomarski differential interference contrast equipped with an Olympus DP72 digital camera. Measurements of spores were analysed in ImageJ v.1_44p (Wayne Rasband, http://imagej.nih.gov/ij). Measurements are presented in micrometres. Means, standard deviation (SD) and range in the parentheses were calculated for each spore dimension. Range of plasmodia size is followed by mean and median in parentheses. For examination of fine structure of myxosporean spores and plasmodia by transmission electron microscopy (TEM), whole urinary bladders as well as samples of their contents and kidney tissue were fixed in cacodylate buffered 3% glutaraldehyde at 4 °C, rinsed in 0.1 M cacodylate buffer and postfixed in 1% osmium tetroxide. After graded acetone dehydration, the samples were embedded in Spurr’s resin. Ultrathin sections were stained with uranyl acetate and lead citrate and examined in a JEOL JEM 1010 electron microscope operating at 80 kV. Images were collected with Megaview II soft paging system using analySIS software.

For histological examination, organs were fixed for 24 h in Davidson fixative, stored in 70% ethanol; samples were routinely dehydrated and embedded into paraffin. Sections were stained by haematoxylin and eosin (HE) and Giemsa. Positive samples were preserved in TNES buffer (10 mM Tris–HCl pH 8, 125 mM NaCl, 10 mM EDTA, 0.5% SDS, 4 M urea) for DNA isolation and selected samples were molecularly characterised by sequencing of rRNA genes.

### 2.3 DNA isolation and PCR

Total DNA was extracted by standard phenol–chloroform method after digestion with proteinase K (100 μg ml^−1^) overnight at 55 °C. The extracted DNA was resuspended in 100 μl of sterile dd H_2_O and kept at 4 °C. SSU rDNA sequences were obtained by PCR using universal eukaryotic ERIB1-ERIB10 primers or by the combination of MyxospecF-ERIB10 and ERIB1-MyxospecR primers (Barta et al., 1997, Fiala, 2006). If the primary PCR failed, the reaction with ERIB primers was followed by nested PCR with combinations of MyxospecF-ERIB10, ERIB1-MyxospecR, and/or MyxospecF-MyxospecR primers. PCRs of the SSU rDNA were carried out in a 25 μl reactions using 1 × Taq buffer, 250 μM of each dNTPs, 10 pmol of each primer, 1 U of Taq-Purple polymerase (Top-Bio, Czech Republic), 1 μl of DNA and sterile dd H_2_O. Cycling parameters for the primary/nested PCR were as follows: denaturation at 95 °C for 3 min, then 30 cycles of amplification at 95 °C for 1 min, 48 °C/50 °C for 1 min, 72 °C for 2/1 min and followed by a 10 min of extension at 72 °C. If above mentioned PCR combinations failed to amplify the desired product TITANIUM Taq DNA polymerase (BD Biosciences Clontech) was used instead of Taq-Purple polymerase. PCRs were conducted in 10 μl reactions with 0.025 U μl^−1^ TITANIUM Taq DNA polymerase, 10 × buffer containing 5 mM MgCl_2_, 0.2 mM of each dNTPs, 0.5 mM of each primer, and 0.5 μl DNA. Cycling parameters for the primary/nested PCR were as follows: denaturation at 95 °C for 2 min, then 30 cycles of amplification at 95 °C for 40 s, 52 °C/56 °C for 40 s, 68 °C for 1 min 40 s/1 min and followed by a 8 min of extension at 68 °C. The 3′ end of the LSU rDNA was obtained using the NLF1050-NLR3284 primer set (Bartošová et al., 2009, Van der Auwera et al., 1994). When these PCRs failed to amplify the desired products, a nested PCR approach with NLF1260-NLR3113 (Bartošová et al., 2009, Van der Auwera et al., 1994) primers was used. The LSU rDNA of *P. minibicornis* failed to amplify with primers listed above but was amplified by nested PCR using primers 28Scer5F1-28Scer5R1 (first PCR) and 28Scer5F2-28Scer5R2 (second PCR) according to Fiala et al. (in prep.). PCRs of the LSU rDNA were carried out in a 25 μl reactions using 1 × LA buffer, 0.5 μl DMSO, 250 μM of each dNTPs, 10 pmol of each primer, LA DNA polymerase (Top-Bio, Czech Republic), 1 μl of DNA, and sterile dd H_2_O. Cycling parameters of LSU rDNA samples in the primary/nested PCR were denaturation at 95 °C for 3 min, then 30 cycles of amplification at 95 °C for 1 min, 50 °C/54 °C for 1 min, 68 °C for 2 min/1 min 40 s and followed by 8 min of extension at 68 °C.

All PCR products were purified using Gel/PCR DNA Fragments Extraction Kit (Geneaid Biotech Ltd., USA). PCR products were sequenced directly or cloned into pDrive Cloning vector (Qiagen, Germany) and transformed into the competent *Escherichia coli* strains XL-1. PCR products or plasmid were sequenced on an ABI PRISM 3130XL automatic sequencer (Applied Biosystems, Czech Republic).

### 2.4 Phylogenetic analyses

The overlapping partial sequences of both SSU and LSU rDNA markers were assembled into the contigs in the SeqMan II program v5.05 (DNASTAR Inc., Madison, Wisconsin). The SSU and LSU rDNA alignments were created in program MAFFT v6.864 (Katoh et al., 2002) using L-INS-i strategy and default parameters. Alignments contain newly obtained sequences and sequences retrieved from GenBank. Highly variable parts of the alignments were determined and excluded in SeaView v4 (Gouy et al., 2010) by Gblocks (Castresana, 2000) using less stringent parameters and slightly adjusted by eye mainly at the beginning and at the end of the alignment.

Five alignments were assembled: SSU rDNA-muc alignment with all newly sequenced myxosporeans and all sequences of taxa within the marine urinary clade available in GenBank (1491 characters) plus the representatives of the other marine clades; LSU rDNA alignment (1987 characters) with all newly sequenced LSU rDNA and those ones available in GenBank; concatenated SSU rDNA-muc + LSU rDNA alignment (3487 characters); SSU rDNA-mar-myxid alignment focused on the marine *Myxidium* clade (1549 characters); and SSU rDNA-cer alignment focused on the *Ceratomyxa* clade (1389 characters). Three myxosporean species from the freshwater lineage were selected as outgroup in the analyses of SSU rDNA-muc alignment and SSU rDNA + LSU rDNA alignment. Outgroups for the SSU rDNA-mar-myxid alignment, SSU rDNA-cer alignment, and LSU rDNA alignment were selected as follows: two species from *Zschokkella* subclade, three ceratomyxids from elasmobranchs and two species from the freshwater lineage, respectively.

Phylogenetic analyses were performed using maximum likelihood (ML), maximum parsimony (MP) and Bayesian inference (BI). ML was done in the RAxML v7.0.3. (Stamatakis, 2006) with GTR GAMMA model of evolution. MP was performed in the PAUP∗ v4.0b10 (Swofford, 2003) with heuristic search with random taxa addition and the TBR swapping algorithm. All characters were treated as unordered, Ts:Tv ratio was set to 1:2 and gaps were treated as missing data. BI was computed in the MrBayes v3.0 (Ronquist and Huelsenbeck, 2003) with the GTR + Γ + I model of evolution. Posterior probabilities were calculated over 1,000,000 generations via two independent runs of four simultaneous Markov chain Monte Carlo chains with every 100th tree saved. Tracer v1.4.1 (Rambaut and Drummond, 2007) was used to set the length of burn-in period. For ML and MP, the bootstrap supports were calculated from 500 replicates. Genetic distances (converted to similarities in %) were computed in PAUP∗ v4.0b10 with default P parameter from the SSU rDNA-muc and SSU rDNA-mar-myxid alignments.

## 3 Results

### 3.1 Findings of myxosporean infections

A total of five (i.e. *M. scorpius*, *G. tricuspis*, *C. harengus*, *B. saida*, *H. platessoides*) out of eight fish species were positive for the presence of Myxozoa (Table 1). 18% out of all dissected fishes were infected by Myxosporea. Two fish were infected with more than one myxosporean: *M. scorpius* with four myxosporeans and *H. platessoides* with two myxosporeans. In this fish species, several concomitant infections occurred (stated in the species descriptions below). The highest prevalence of myxosporean infection was observed in *H. platessoides* and *B. saida* (Table 1).

**Table 1:** List of myxosporeans, hosts, site of infection (gb = gall bladder, ub = urinary bladder), prevalence (number of infected organs/number of analysed organs) and measurements of species found on Svalbard. Spore: Length (*L*), Width (*W*), Thickness (*T*), Polar capsules (PCs). All measurements in μm.

Myxosporean species	Host	Site of infection	Prevalence	Spores			PCs	Plasmodia	References
				*L*	*W*	*T*			
*Ceratomyxa porrecta*	*Myoxocephalus scorpius*	gb	4% (3/79)	2.9 ± 0.4 (2.3–3.2)	29.1 ± 4.8 (25.5–34.4)	–	1.9 ± 0.2 × 1.7 ± 0.2	3.9 ± 0.5 × 10.2 ± 2.2 (3.5–13.8)	This study
				4–5	23–34	–	3 × 3	–	Dogiel (1948)
*Myxidium gadi*		gb	6% (5/79)	11.3 ± 0.1 (11.2–11.4)	5.3 ± 2.0 (3.9–6.7)	–	3.4 ± 0.5 × 2.8 ± 0.7	–	This study
				8.5–14	4–7.5	–	4.5–4.7	–	Géorgevitch (1916) (quoted from Shulman (1966))
*Myxidium finnmarchicum*		gb	7% (6/79)	15.3 ± 1.6 (13.1–17.8)	9.2 ± 1.3 (7.2–10.1)	9 ± 0.5 (8.4–9.7)	4.8–6.4 × 3.2–4.8	24.0 ± 3.2 (22.2–27.7) × 27.5 ± 3.6 (25.0–31.6)	This study
				17.6–22.4	6.4–6.9	–	4.8–6.4 × 3.2–4.8	30–36 × 40–50	MacKenzie et al. (2010)
*Sinuolinea arctica*		ub	10% (5/48)	15.7 ± 0.9 (14.7–16.6)	15.4 ± 0.8 (14.2–16.6)	16.1 ± 2.9 (14.0–18.1)	5.1 ± 0.3 × 5.1 ± 0.3	21.8 ± 4.7 (16.6–31.3) × 27.4 ± 7.0 (18.0–39.5)	This study
*Parvicapsula petuniae*	*Gymnocanthus tricuspis*	ub, kidney	9% (2/22)	11.0 ± 0.7 (9.9–12.3)	7.9 ± 0.6 (7.4–8.3)	8.7 ± 2.1 (6.7–11.9)	3.6 ± 0.2 × 2.8 ± 0.3	–	This study
*Zschokkella siegfriedi*	*Boreogadus saida*	kidney	43% (6/14)	17.4 ± 0.7 (16.7–18.2)	10.5 ± 1.2 (9.2–11.6)	9.8 ± 0.7 (8.5–11.0)	5.2 ± 0.3 × 5.1 ± 0.3	16.6 ± 4.2(11.5–24.0) × 20.8 ± 5.3 (14.0–25.1)	This study
*Parvicapsula irregularis*	*Hippoglossoides platessoides*	ub, kidney	44% (4/9)	11.0 ± 0.7 (11.0–15.1)	7.9 ± 0.6 (6.1–10.4)	8.7 ± 2.1 (7.4–9.0)	3.6 ± 0.2 × 2.8 ± 0.3	10.6 ± 0.7 (10.1–11.1) × 13.3 ± 1.5 (12.3–4.3)	This study
				8.0–11.0 (mean 10.6)	6.0–9.0 (mean 7.1)	–	2.2	15–20 × 20–25	Kabata (1962)
*Schulmania aenigmatosa*		ub	22% (2/9)	20.3 ± 1.6 (17.2–22.9)	16. 9 ± 1.6 (15.0–19.5), with wings 17.4 ± 1.5 (16.0–19.6)	16.4 ± 6.3 (14.9–18.0)	6.7 ± 0.6 × 6.3 ± 0.5	29.3 ± 3.3 (27.2–31.2) × 30.8 ± 3.4 (28.0–37.4)	This study
				19.9–23.1	11.9–13.3	11.9–16.0	5.9–6.7	26.6–42.0 × 31.9–55.9	Kovaleva et al. (1983)
*Latyspora*-like organism	*Clupea harengus*	kidney	14% (9/66)	10.7 ± 0.7 (9.2–11.2)	22.6 ± 1.6 (21.2–26.2)	20.7 ± 2 (17.7–23.3)	7.8 ± 0.4 × 4.7 ± 0.4	28.4 ± 4.1 (23.1–33.7) × 29.8 ± 3.2 (25.3–32.8)	This study

We obtained 9 SSU rDNA sequences of *Parvicapsula irregularis*, *P. petuniae*, *Zschokkella siegfriedi*, *Sinuolinea arctica*, *Schulmania aenigmatosa*, *Latyspora*-like organism, *Ceratomyxa porrecta*, *Myxidium gadi*, and *M. finnmarchicum*. We obtained 6 LSU rDNA sequences of *P. petuniae*, *P. minibicornis* (from *Gasterosteus aculeatus*; Oregon, USA), *Latyspora*-like organism, *P. irregularis*, *S. arctica*, and *S. aenigmatosa*.

### 3.2 Myxosporean species

#### 3.2.1 Additional data on described species

**Fig. 1:**
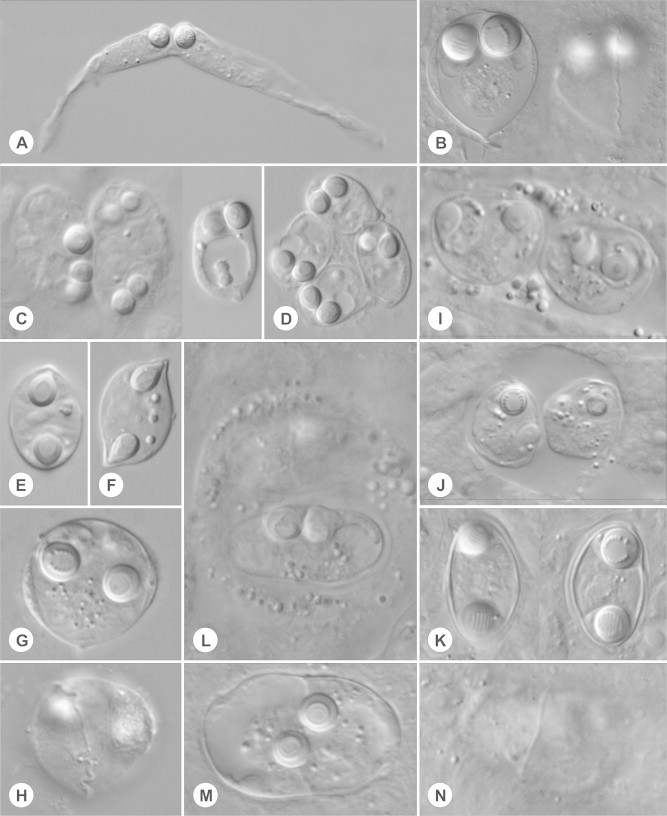
Mature spores and plasmodia. (A-N) Myxospores and myxosporean plasmodial stages as seen in Nomarski differential interference contrast. Measurements are listed in Table 1. (A) Mature spore of *Ceratomyxa porrecta*. (B) Spores of *Schulmania aenigmatosa* with focus on polar capsules (left) and sinuous valve suture (right). (C) Plasmodial stages (left) and mature spore of *Parvicapsula irregularis* (right). (D) Mature spores of *Parvicapsula petuniae.* (E) Mature spore of *Myxidium gadi*. (F) Mature spore of *Myxidium finnmarchicum*. (G, H) Spores of *Sinuolinea arctica* in frontal (G) and sutural (H) view. (I, J) Plasmodial stages of *Zschokkella siegfriedi*. (K) Mature spores of *Zschokkella siegfriedi.* (L) Plasmodial stage of *Latyspora*-like organism. (M, N) *Latyspora*-like organism spores with focus on polar capsules and part of valve suture, respectively.

***Ceratomyxa porrecta*** Dogiel, 1948 (Fig. 1A).

*Type host: Gymnocanthus herzensteini* Jordan and Starks, 1904.

*Other hosts*: *M. scorpius* (Linnaeus, 1758), shorthorn sculpin, average standard length 18.9 cm; *Bero elegans* (Steindachner, 1881); *Myoxocephalus brandtii* (Steindachner, 1867).

*Type locality*: Peter the Great Bay, Japan Sea.

*Other locality*: Greenland Sea, part of the Billefjorden, Isfjorden, Petunia Bay in the central part of Svalbard archipelago (78° 69′ N, 16° 53′ E).

*Description of sporogonic stages*: disporic plasmodia with filopodia; for dimensions see Table 1.

*Description of myxospores*: crescent shape with markedly elongated shell valves; PCs with a straight central shaft of the filament, located close to the suture line in a plane perpendicular to it; posterior angle 220°; for dimensions see Table 1.

*Localization of sporogonic stages*: coelozoic, gall bladder.

*Prevalence*: 4% (3 of 79 gall bladders; 1 sample co-infected with *Myxidium finnmarchicum*).

*Pathology*: no material available for evaluation the species pathogenicity.

*Materials deposited*: DNA sample (nr. 1373) stored in −80 °C in the Laboratory of Fish Protistology, Institute of Parasitology, BC ASCR; SSU (GenBank accession No. KF874235) rDNA sequence.

*Remarks: Ceratomyxa porrecta* has identical spore shape with original description of *C. porrecta* (Dogiel, 1948). Measurements differences (see Table 1): PCs are remarkably larger (3 × 3 μm), and spore length is longer (4.5 μm) in Dogiel’s description of *C. porrecta* contrary to our measurements of *C. porrecta* from this study (PC 1.9 × 1.7 μm; spore length 2.9 μm). The type host of *C. porrecta* is *G. herzensteini* Jordan and Starks, 1904. We suggest that *M. scorpius* is another host for *C*. *porrecta*. Although our material does not originate from the type host of *C. porrecta* and there is no sequence data for *C. porrecta*. We assign our molecular and morphological findings to *C. porrecta* based on identical spore morphology and on the close genetic relationship of hosts *M. scorpius* and *M. brandtii* (Knope, 2013).

***Myxidium gadi*** Georgévitch, 1916 (Fig. 1E).

*Type host: Pollachius pollachius* (Linnaeus, 1758), pollack.

*Other hosts*: *M. scorpius* (Linnaeus, 1758), shorthorn sculpin, average standard length 18.5 cm; *G. morhua* Linnaeus, 1758, Atlantic cod; *Pollachius virens* (Linnaeus, 1758), saithe; *Merlangius merlangius* (Linnaeus, 1758), whiting; *Melanogrammus aeglefinus* (Linnaeus, 1758), haddock; *Pleuronectes flesus* Linnaeus, 1758, European flounder; *Solea solea* (Linnaeus, 1758), common sole.

*Type locality*: Roscoff, off France coast.

*Other localities:* Barents Sea, White Sea, Atlantic Ocean: off Canada coast, Greenland Sea, part of the Billefjorden, Isfjorden, Petunia Bay in the central part of Svalbard archipelago (78° 69′ N, 16° 53′ E).

*Description of sporogonic stages*: plasmodia not observed in our material.

*Description of myxospores*: fusiform shape with pointed ends; pyriform PCs at each end of the spore; for dimensions see Table 1.

*Localization of sporogonic stages:* coelozoic, gall bladder.

*Prevalence*: 6% (5 of 79 gall bladders).

*Pathology*: no material was available for evaluation the species pathogenicity.

*Materials deposited*: DNA sample (nr. 1320) stored in −80 °C in the Laboratory of Fish Protistology, Institute of Parasitology, BC ASCR; SSU (GenBank accession No. KF874236) rDNA sequence.

*Remarks: Myxidium gadi* has a wide host species spectrum and has been reported from five gadids and two flatfish (MacKenzie et al., 2010, Shulman et al., 1966). *M. scorpius* is a new host for *M. gadi*, broadening its host spectrum in the family Cottidae, as spore measurements of our material from shorthorn sculpin basically correspond to the original description of *M. gadi* from *P. pollachius* (Table 1). Moreover, *M. gadi* from shorthorn sculpin and *M. gadi* from haddock are genetically highly similar (98.8%; Supplementary Fig. 2A, Table 2). Moreover intraspecific variability based on partial SSU rDNA sequence of *M. gadi* is high, on the edge of the border resolving two species. This variability can be driven by wide host spectrum with intermixing infrapopulations of *M. gadi*. Infrapopulation of *M. gadi* on the edge of distribution area can be for some period of time without any gene flow with the rest of infrapopulations. Four *Myxidium* species were found in cottids: *M. scorpii* Schulman-Albowa, 1950, described from atypical infection site in the urinary bladder of *M. scorpius*; *M. arcticum* Zhukov, 1962 described from *Myoxocephalus axillaris*; *M. japonicum* Dogiel, 1948 described from *Myoxocephalus brandtii;* and *M. myoxocephali* Fantham, Porter and Richardson, 1940 described from *Myoxocephalus octodecemspinosus*. *Myxidium myoxocephali* appears to be identical with *M. incurvatum* based on their similar morphology, morphometrics and distribution area, although Fantham et al. (1940) noted that the parasite was larger than *M. incurvatum* (Khan et al., 1986) and both myxosporeans also differ in host species and molecularly. Generally, marine *Myxidium* species clustering within the marine *Myxidium* clade have fusiform or S-shape spores. The spore measurements and shape of *M. gadi* correspond to those of *M. scorpii* described from the same host but they differ in tissue tropism and PCs size. *Myxidium scorpii* described from an atypical infection site in the urinary bladder has slightly smaller PCs (1.8–2.0 μm) than *M. gadi* (3.4 × 2.8 μm) originating from gall bladder of *M. scorpius* (Table 1).

***Myxidium finnmarchicum*** MacKenzie et al., 2010 (Fig. 1F).

*Type host*: *Merlangius merlangus* (Linnaeus, 1758), Whiting.

*Other host*: *M. scorpius* (Linnaeus, 1758), Shorthorn sculpin, average standard length 16.5 cm;

*Type locality*: off Sørøya, North Norway (70° 47′ N, 22° 58′ E).

*Other localities:* Greenland Sea, part of the Billefjorden, Isfjorden, Petunia Bay in the central part of Svalbard archipelago (78° 69′ N, 16° 53′ E).

*Description of sporogonic stages*: spherical disporic plasmodia; for dimensions see Table 1.

*Description of myxospores*: sigmoid shape with pointed ends; fine transverse ridges; pyriform PCs at each end of the spore, for dimensions see Table 1.

*Localization of sporogonic stages:* coelozoic, gall bladder.

*Prevalence*: 7% (6 of 79 gall bladders; 2 samples co-infected with *Ceratomyxa porrecta*).

*Pathology*: no material available for evaluation the species pathogenicity.

*Materials deposited*: DNA sample (nr. 1610) stored in −80 °C in the Laboratory of Fish Protistology, Institute of Parasitology, BC ASCR; SSU rDNA sequence (GenBank accession No. KF874237).

*Remarks: M. scorpius* is a new host for *M. finnmarchicum*, broadening its host species spectrum in the family Cottidae. *Myxidium finnmarchicum* was described with 4–6 fine longitudinal striations (MacKenzie et al., 2010) which were not observed in this study. On the other side, we observed fine transverse ridges on *M. finnmarchicum* spores under the light microscope. However, scanning electron micrographs are required for re-evaluation of the spore surface. SSU rDNA sequences of the myxosporean from our three samples were almost identical with SSU rDNA data of *M. finnmarchicum*. *Myxidium finnmarchicum* has a similar spore shape as *M. gadi* but differs in larger spore size and genetic similarity is 94.4% (Supplementary Table 2).

**Fig. 2:**
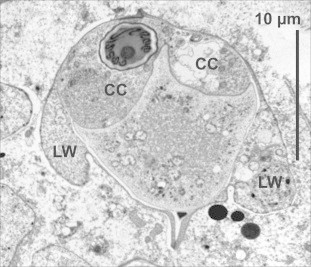
Ultrathin section of almost mature spore of *Schulmania aenigmatosa* with lateral wings (LW) typical for the genus. CC capsulogenic cell, CC with polar capsule (left).

**Fig. 3:**
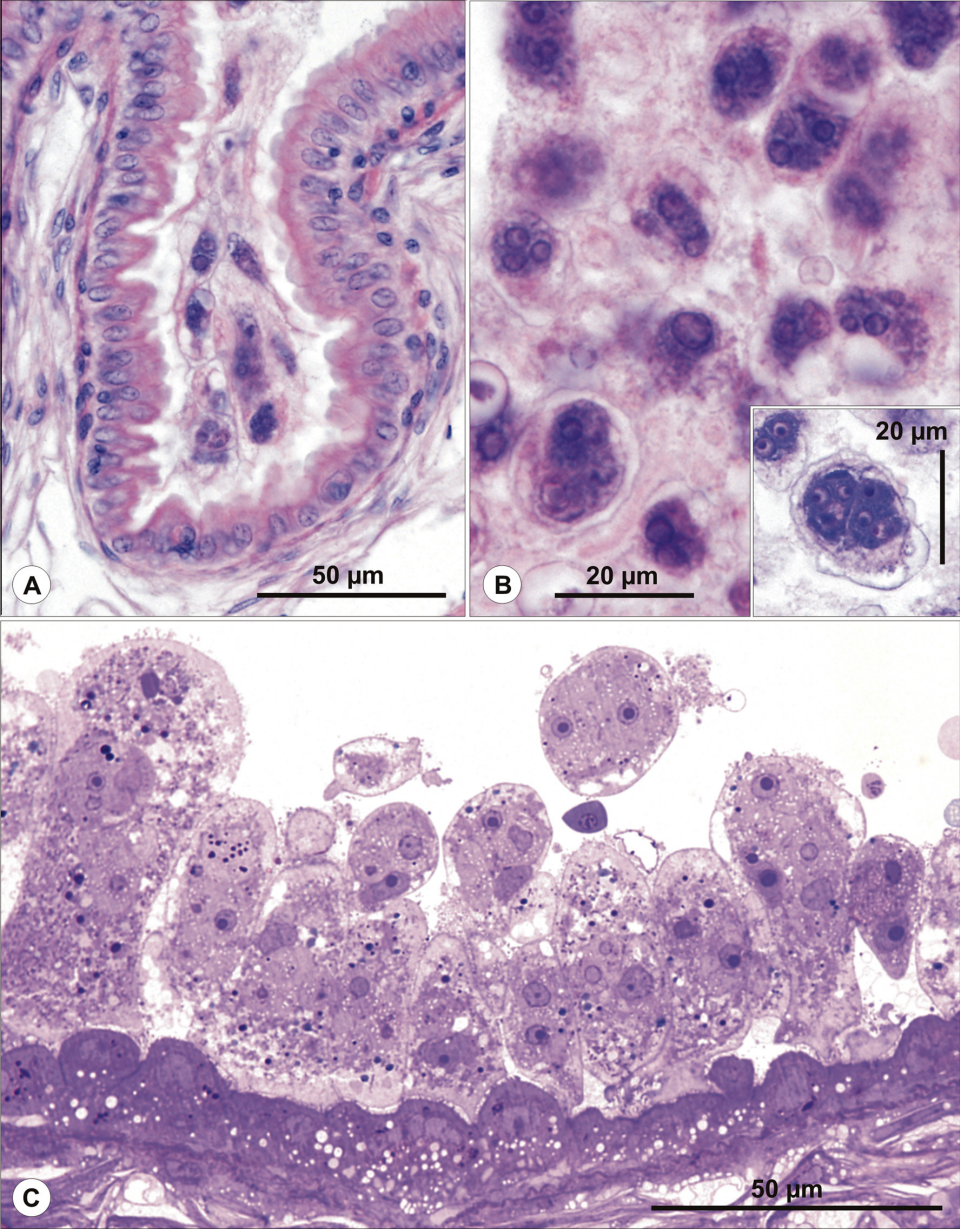
Histology of *Schulmania aenigmatosa* infection. (A-C) *Schulmania aenigmatosa* infection in excretory system of *Hippoglossoides platessoides*. (A) Early plasmodial stages localised in ureter as seen in histological section stained with HE. (B) Advanced plasmodial stages filling urinary bladder. Giemsa stained stage (inserted). (C) Semithin section stained with toluidine blue documents numerous plasmodial stages attached to the wall of urinary bladder. (For interpretation of the references to colour in this figure legend, the reader is referred to the web version of this article.)

***Schulmania aenigmatosa*** (Kovaleva et al., 1983) (Fig. 1B, Fig. 2, Fig. 3).

*Type host*: *H. platessoides* (Fabricius, 1780) American plaice, average standard length 10.5 cm.

*Other hosts*: *Hippoglossoides robustus* Gill and Townsend, 1897, Bering flounder; *Hippoglossoides elassodon* Jordan and Gilbert, 1881, Flathead sole.

*Type locality*: south off the Labrador.

*Other localities:* Sea of Okhotsk, Chukchi Sea, Bering Sea, Greenland Sea, part of the Billefjorden, Isfjorden, Petunia Bay in the central part of Svalbard archipelago (78° 69′ N, 16° 53′ E).

*Description of sporogonic stages*: spheric or plane (oval in cross section) disporic plasmodia; ectoplasm separated from endoplasm; for dimensions see Table 1.

*Description of myxospores*: spores notably large, inversely pyramidal in sutural view with pointed posterior pole; suture line waved; lateral wings partially visible in the light microscope and clearly in TEM, lateral wings on spore present as pocket-like extensions separated from the spore body by membrane; PCs in posterior pole apposed closely to each other and discharging forward in the direction slightly toward the axis of the spore, 7 coils of polar filament; for dimensions see Table 1.

*Localization of sporogonic stages*: coelozoic, urinary bladder.

*Prevalence*: 22% (2 of 9 urinary bladders; 2 samples co-infected with *P. irregularis*).

*Pathology*: high numbers of rodlet cells observed in epithelium of heavily infected segments of renal tubules (Fig. 3); even seen in early infections but not present in epithelium of collecting ducts and urinary bladder.

*Materials deposited*: DNA sample (nr. 1415) stored in −80 °C, paraffin blocks nrs. 786/10, 819/10, 900/10 and blocks in resin nr. 550i stored in the Laboratory of Fish Protistology, Institute of Parasitology, BC ASCR; SSU (GenBank accession No. KF874233) and LSU (GenBank accession No. KF874228) rDNA sequences.

*Remarks:* The spores of *S. aenigmatosa* from our material were wider in frontal view compared to the original description of Kovaleva et al. (1983) (12 μm vs. 17 μm). *Schulmania aenigmatosa* is very similar by its spore size with *S. ovale*; the type species of the genus *Schulmania*. Mainly four longitudinal keel-like stiff membranes and wing shaped extensions were partially visible by light microscopy and clearly visible in TEM (Fig. 2). The lateral wings are one of the most characteristic features of the genus *Schulmania*. However, due to our rare observation of this feature one can deduce that this structure changes during its maturation as observed in other myxosporeans e.g. immature spores of *Bipteria formosa* have empty-looking pockets at each spore side which detach posteriorly in maturing spores and later open thus releasing their content (Karlsbakk and Køie, 2009). *Schulmania aenigmatosa* is the first sequenced member of the genus *Schulmania*.

***Parvicapsula irregularis*comb. nov.** (Kabata, 1962) (Fig. 1C).

*Synonyms: Sphaerospora irregularis*Kabata, 1962; *Myxoproteus irregularis* (Kabata, 1962); *Ortholinea irregularis* (Kabata, 1962).

*Type host*: *H. platessoides* (Fabricius, 1780), American plaice (syn. *Drepanopsetta platessoides* Fabricius, 1780); average standard length 10.6 cm.

*Other hosts*: unknown.

*Type locality*: Northern North Sea.

*Other localities:* Greenland Sea, part of the Billefjorden, Isfjorden, Petunia Bay in the central part of Svalbard archipelago (78° 69′ N, 16° 53′ E).

*Description of sporogonic stages*: disporic plasmodia of various shapes i.e. round, oval or irregular with protrusions; pseudopodia with rounded ends; for dimensions see Table 1.

*Description of myxospores*: spore shape roughly pyriform with considerable degree of irregularity, widest diameter of spore about middle of the long axis, narrowing somewhat towards the poles, particularly towards the anterior pole; slightly flattened in the sutural plane; spherical PCs close together located anteriorly; single sporoplasm occupying more than three quarters of the spore, sporoplasm with two nuclei; for dimensions see Table 1.

*Localization of sporogonic stages:* coelozoic, renal tubules, urinary bladder.

*Prevalence*: 44% (4 of 9 urinary bladders; 2 samples co-infected with *Schulmania aenigmatosa*).

*Pathology*: no material available for evaluation the species pathogenicity.

*Materials deposited*: DNA sample (nr. 1376) stored in −80 °C in the Laboratory of Fish Protistology, Institute of Parasitology, BC ASCR; SSU (GenBank accession No. KF874229) and LSU (GenBank accession No. KF874226) rDNA sequences.

*Remarks:*Kabata (1962) described *Sphaerospora irregularis* from American plaice in northern North Sea. This species was later assigned to other genera: *Myxoproteus* (Gaevskaya and Kovaleva, 1984), and *Ortholinea* (Arthur and Lom, 1985). After re-examination, Køie et al. (2007b) suggested *S. irregularis* may belong to *Parvicapsula*. Despite *S. irregularis* was reported from another host, *Pleuronectes platessa* (MacKenzie et al., 1976), this report most probably corresponds to *Parvicapsula bicornis* later described from this host (Køie et al., 2007b). Unfortunately, the report of “*S. irregularis*” by MacKenzie et al. (1976) lacked sufficient morphological documentation and comparison with similar species. Therefore, *P. bicornis* from *P. platessa* was regarded as syn. part. of *S. irregularis* (Køie et al., 2007b). Since this species is now re-examined and molecularly characterised we claim that *P. bicornis* and the re-described *P. irregularis* are two morphological and molecularly different species. *S. testicularis* as the closest relative of *P. irregularis* has a wider and thicker spore.

#### 3.2.2 Description of new taxa

**Fig. 4:**
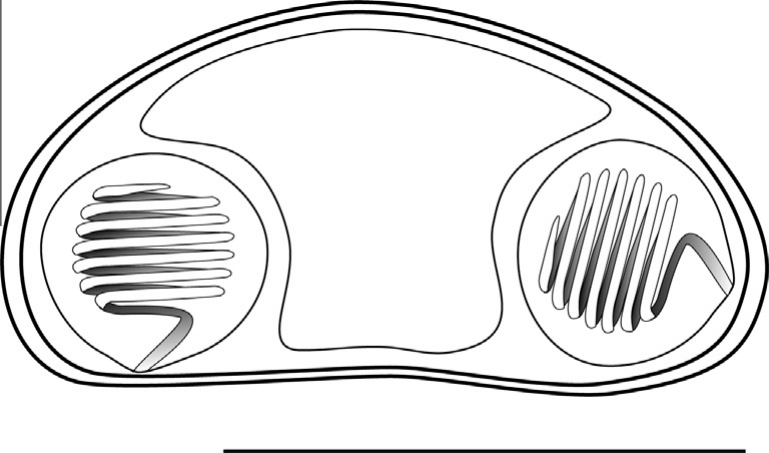
Line drawing of *Zschokkella siegfriedi*, sutural view. Scale bar = 10 μm.

**Fig. 5:**
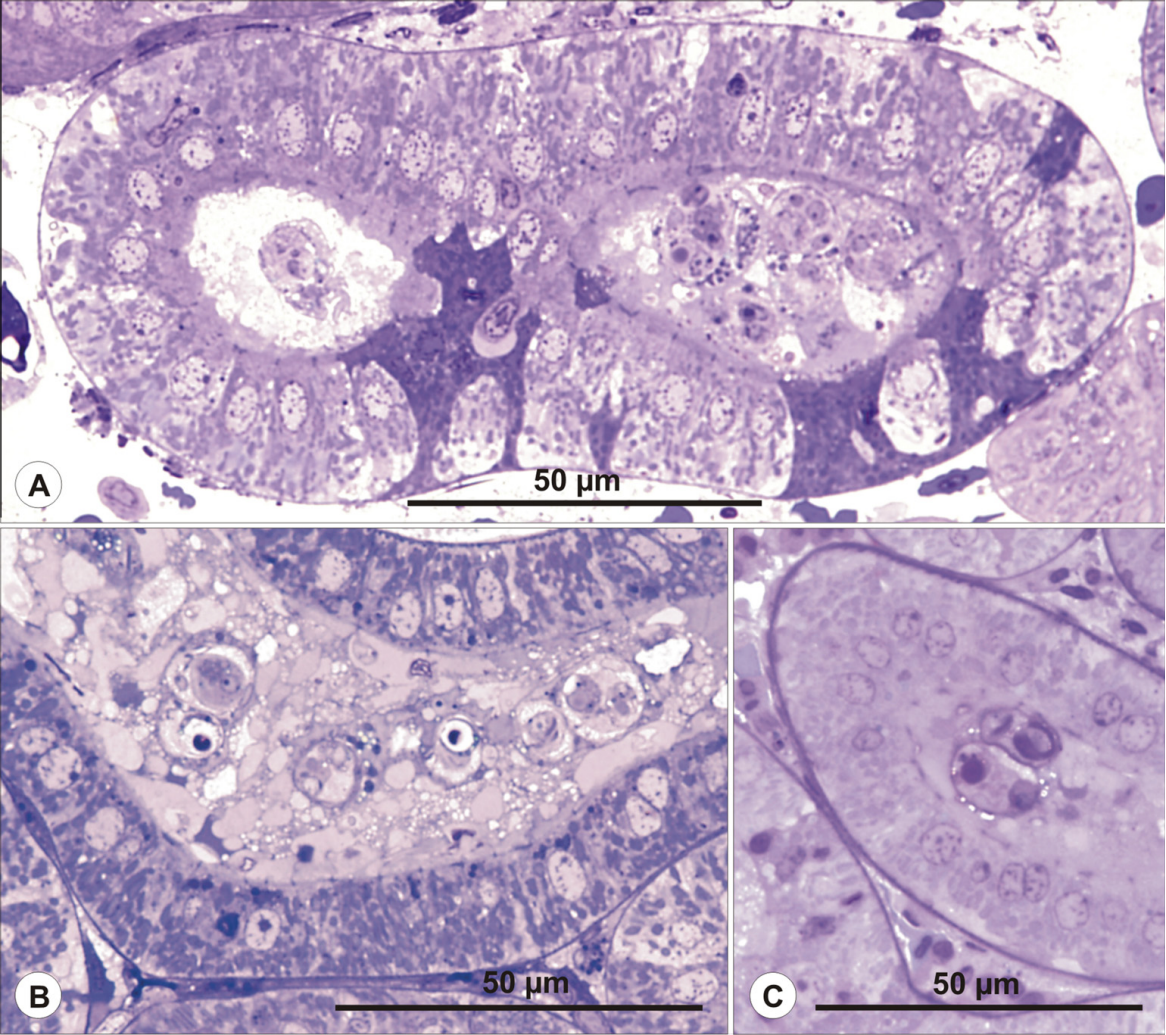
Histology of *Zschokkella siegfriedi* infection. (A–C) *Zschokkella siegfriedi* infection in renal tubules of *Boreogadus saida* as seen in semithin sections stained with toluidine blue. (A) Infected segment of renal tubule with plasmodial stages in its lumen and densely stained cells in its epithelial lining. (B) Advanced plasmodial stages and amorphous material completely filling the lumen of renal tubule. All epithelial cells are densely stained. (C) Almost mature spores localised in the lumen of renal tubule. (For interpretation of the references to colour in this figure legend, the reader is referred to the web version of this article.)

**Fig. 6:**
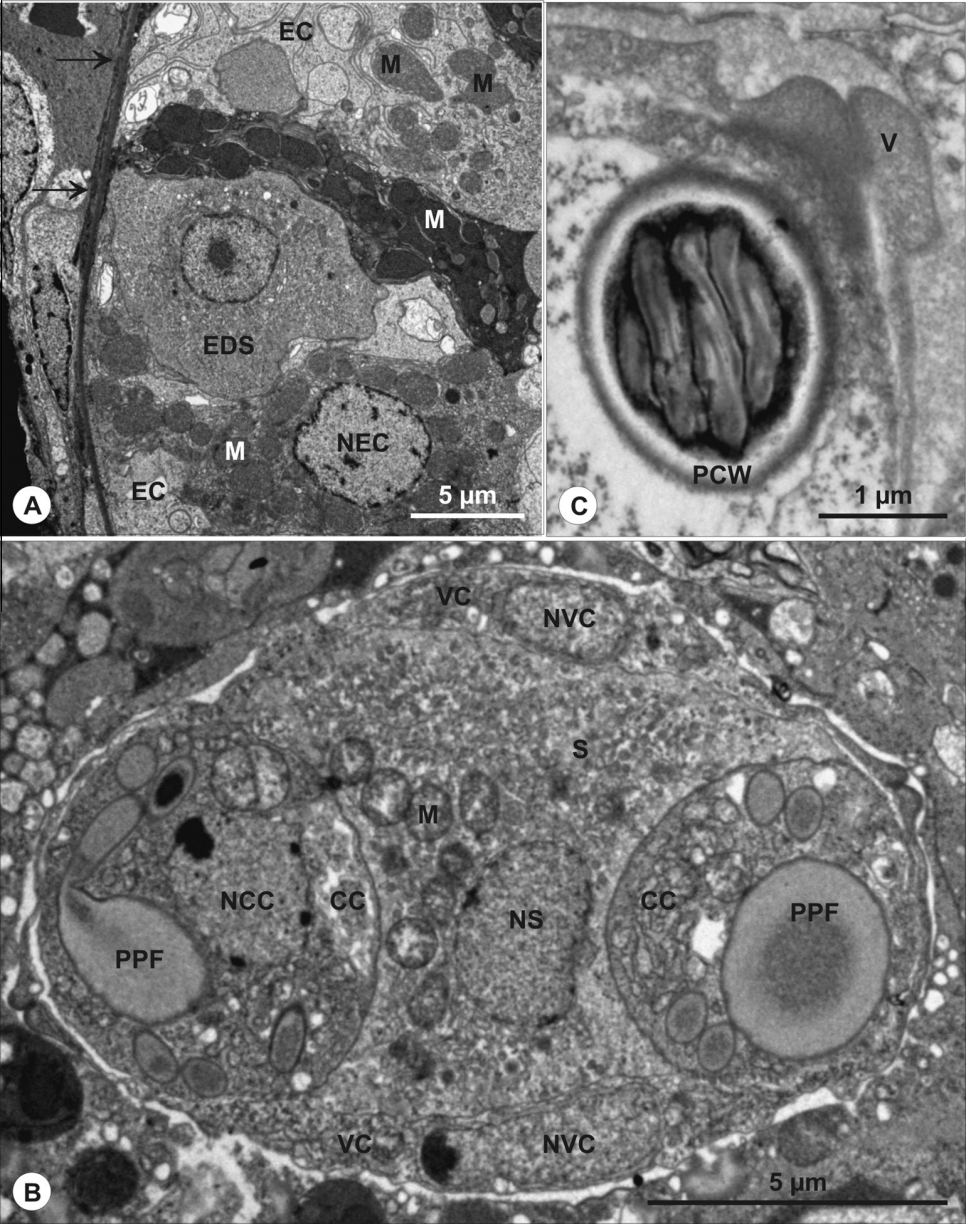
Ultrastructure of *Zschokkella siegfriedi* infection. (A–C) Details of ultrastructure of *Zschokkella siegfriedi* as seen in transmission electron microscope. (A) Early developmental stage (EDS) localised within epithelium of renal tubule. Epithelial cells (EC) differ substantially in electron-density due to differences in density of mitochondria. Arrows mark basal membrane of renal tubule, NEC nucleus of epithelial cells, M mitochondria. (B) Almost mature spore in longitudinal section. VC valvogenic cell, NVC nucleus of VC, CC capsulogenic cells, NCC nucleus of CC, S sporoplasm, NS nucleus of S, PPF primordium of polar filament, M mitochondria. C. Valves (V), the polar capsule wall (PCW), and some sections of the polar filament coils.

***Zschokkella siegfriedi*n. sp.** (Fig. 4, Fig. 5, Fig. 6, Fig. 4, Fig. 5, Fig. 6).

Family Myxidiidae Thélohan, 1892.

Genus *Zschokkella* Auerbach, 1910.

*Type host*: *B. saida* (Lepechin, 1774), Polar cod (officially accepted common name; commonly used name Arctic cod for *B. saida* is valid for *Arctogadus glacialis* (Peters, 1872) (Froese and Pauly, 2013); average standard length 14.4 cm.

*Other host:* unknown.

*Type locality*: Greenland Sea, part of the Billefjorden, Isfjorden, Petunia Bay in the central part of Svalbard archipelago (78° 69′ N, 16° 53′ E).

*Other localities:* none.

*Description of sporogonic stages*: plasmodia mostly di-, rarely polysporic; round to oval in shape; clear differentiation between smooth ectoplasm and granular endoplasm; for dimensions see Table 1.

*Description of myxospores*: shape of spores considerably variable, from spores with one side vaulted appearing almost rounded triangular to spores of ellipsoidal shape; suture line irregularly oblique, two shell valves completely asymmetrical; subspherical to spherical PCs located in the spore ends and discharging to opposite sides parallel with axis of the spore from the apical view, 7 coils of polar filament; for dimensions see Table 1.

*Localization of sporogonic stages:* coelozoic, renal tubules.

*Prevalence*: 43% (6 of 14 kidney samples).

*Pathology*: regressive changes of importance developed in the epithelial cells of infected renal tubules manifested as pronounced changes of staining properties of individual cells in semithin sections; mitochondria with various degrees of mitochondrial electron-density suggestive of necrotic changes revealed in ultrathin sections (Fig. 5, Fig. 6).

*Materials deposited*: DNA sample (nr. 1608) stored in −80 °C and blocks in resin nrs. 541a and 543a in the Institute of Parasitology, Laboratory of Fish Protistology, BC ASCR; SSU rDNA sequence (GenBank accession No. KF874231).

*Etymology:* The species name of *Z. hildae*, type species of the genus *Zschokkella*, refers to Hilda (a shorten version of the German name) used by author Auerbach (1910) in honour of his wife. We name *Z. siegfriedi* n. sp. according to the German heroic poem “The Song of Nibelungs” with the lovers Siegfried and Kriemhilda (Hilda) reflecting the close phylogenetic relationship between *Z. hildae* and our new species.

*Remarks:* We found *Zschokkella siegfriedi* from the kidney of polar cod to be genetically distinct (2.8% of dissimilarity) (Supplementary Table 1) from *Z. hildae* SSU rDNA sequence from *G. morhua*. *Zschokkella hildae*, the type species of the genus *Zschokkella*, typically infects fish from the family Gadidae and was previously reported from *B. saida* without providing any molecular data (Aseeva, 2002, Køie, 2009). In the light of the new data, we suggest *B. saida* was most likely either infected with *Z. siegfriedi* in the report of Aseeva (2002) or this host is susceptible for both *Z. hildae* and *Z. siegfriedi* species. The spores of *Z. hildae* possess some degree of pleiomorphy during maturation; morphologically, *Z. hildae* and *Z. siegfriedi* are indistinguishable. However, *Z. hildae* was found to infect the host’s urinary bladder and collecting duct of the kidney, unlike *Z. siegfriedi* which develops in the upper excretory system and the renal tubules. Nevertheless, we expect *Z*. *siegfriedi* to infect also urinary bladder as reported for *Z*. *hildae* since we were not able to cheque the urinary bladder of *B. saida*. We determined that *Z. siegfriedi* is a distinct species based on biology and genetics; biologically, *Z. siegfriedi* has (i) significant genetic difference based on SSU rDNA; (ii) localization of sporogonic stages in renal tubules vs collecting duct; (iii) different but very closely related host species to that of *Z. hildae*.

**Fig. 7:**
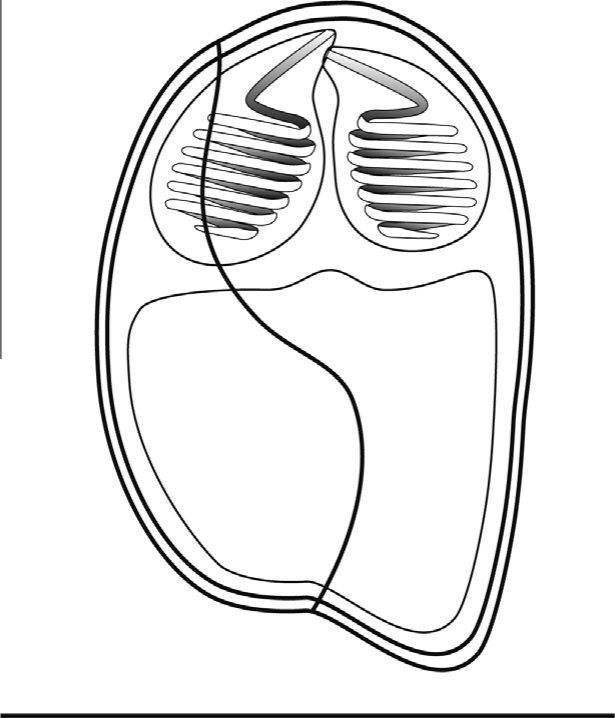
Line drawing of *Parvicapsula petuniae*, sutural view. Scale bar = 10 μm.

***Parvicapsula petuniae*n. sp.** (Fig. 1D, Fig. 7).

Family Parvicapsulidae Shulman, 1953.

Genus *Parvicapsula* Shulman, 1953.

*Type host*: *G. tricuspis* (Reinhardt, 1830), Arctic staghorn sculpin; average standard length 13.9 cm.

*Other hosts*: unknown.

*Type locality*: Greenland Sea, part of the Billefjorden, Isfjorden, Petunia Bay in the central part of Svalbard archipelago (78° 69′ N, 16° 53′ E).

*Other localities:* none.

*Description of sporogonic stages*: disporic plasmodia, early plasmodia subspherical to oval, sometimes with filopodial projections; plasmodia located in renal tubules; for dimensions see Table 1.

*Description of myxospores*: spores asymmetrical with somewhat curved and wavy suture line, ellipsoidal in frontal view; two pyriform PCs of equal size; closely apposed, discharging in the same apical direction, 8 coils of polar filament; single distinct binucleate sporoplasm; measurements see Table 1.

*Localization of sporogonic stages:* coelozoic, renal tubules, urinary bladder.

*Prevalence*: 9% (2 of 22 kidney samples and of 17 urinary bladders).

*Pathology*: No material was available for evaluation the species pathogenicity.

*Materials deposited*: DNA sample (nr. 1423) stored in −80 °C in the Laboratory of Fish Protistology, Institute of Parasitology, BC ASCR; SSU (GenBank accession No. KF874230) and LSU (GenBank accession No. KF874223) rDNA sequences.

*Etymology:* specific name refers to the type locality Petunia Bay.

*Remarks:* This is the first report of a *Parvicapsula* species from *G. tricuspis*. The shape and size of *P. petuniae* spores are similar to the asymmetrical spores of *P*. *hoffmani* infecting the intestinal epithelium of mullet (India) (Dorothy and Kalavati, 1993) and even more similar to *P. karenii* infecting the urinary bladder of a flatfish from the Yellow Sea (Zhao et al., 2000). Nevertheless, the above mentioned morphologically similar *Parvicapsula* species differ in their host species preference and with different distributions from *P. petuniae* thus considering it to be a distinct species.

**Fig. 8:**
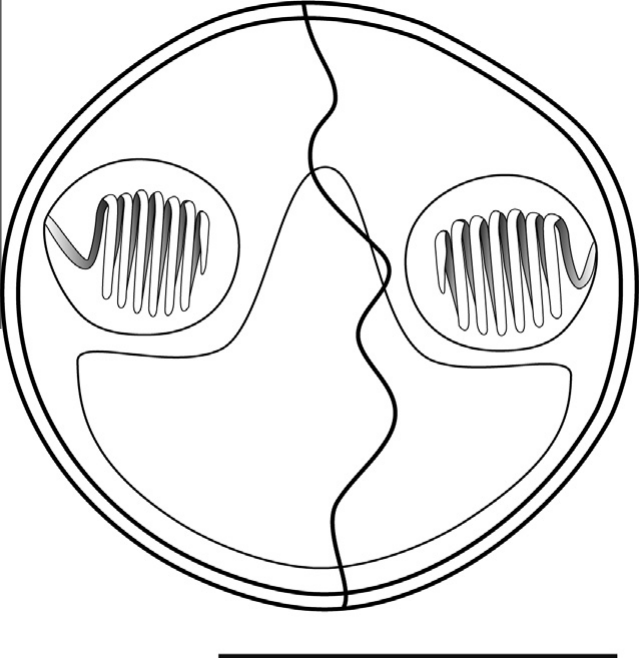
Line drawing of *Sinuolinea arctica*, sutural view. Scale bar = 10 μm.

***Sinuolinea arctica*n. sp.** (Fig. 1G, H, Fig. 8).

Family Sinuolineidae.

Genus *Sinuolinea* Davis, 1917.

*Type host*: *M. scorpius* (Linnaeus, 1758), Shorthorn sculpin; average standard length 18.7 cm.

*Other hosts*: unknown.

*Type locality*: Greenland Sea, part of the Billefjorden, Isfjorden, Petunia Bay in the central part of Svalbard archipelago (78° 69′ N, 16° 53′ E).

*Other localities:* none.

*Description of sporogonic stages:* mostly disporic, rarely polysporic spherical plasmodia; freely floating in urine; spores maturing either inside the plasmodium or in pansporoblasts which are as a whole separated from the plasmodium and where spores subsequently undergo complete maturation; for dimensions see Table 1.

*Description of myxospores*: spores spherical with protrusive sinuous suture line twisted in its axis; valves with smooth surface; two spherical PCs of equal size, separated from one another and discharging sideways, 7 coils of polar filament; spores with a single distinct sporoplasm; for dimensions see Table 1.

*Localization of sporogonic stages:* coelozoic; urinary bladder.

*Prevalence*: 10% (5 of 48 urinary bladders).

*Pathology*: unknown.

*Materials deposited*: DNA sample (nr. 1317) stored in −80 °C in the Laboratory of Fish Protistology, Institute of Parasitology, BC ASCR; SSU (GenBank accession No. KF874232) and LSU (GenBank accession No. KF874227) rDNA sequences.

*Etymology:* species name refers to the geographic origin in Arctic.

*Remarks: Sinuolinea arctica* is the first *Sinuolinea* species described from *M. scorpius*. Its size and morphology are similar to those of the type species *S. dimorpha*, but the spore of *S. arctica* is slightly bigger (14.8–15 μm vs. 15.4–16 μm) (Dyková et al., 2013). *Sinuolinea* sp. from urine of *M. scorpius* was previously reported by Lom (1984) and has identical morphology to *S. arctica*. However, dimensions of *Sinuolinea* sp. are significantly larger (L 22.9 μm and W 20.7 μm) than of *S. arctica* (L 15.7 ± 0.9 and W 15.4 ± 0.8). Assigning taxonomic status of *Sinuolinea* sp. would require molecular characterisation. *Myxoproteus myoxocephali* Fantham, 1940 (family Sinuolineidae) was described from gall bladder of *M. scorpius*. However, infecting gall bladder, which is not a typical site of infection of sinuolineid species, and poor morphological description of *M. myoxocephali* puts doubt on correct systematic position of this species.

#### 3.2.3 Characterization of new organism

**Fig. 9:**
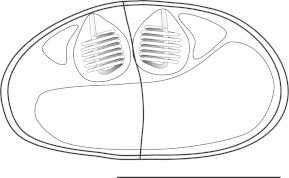
Line drawing of *Latyspora*-like organism, sutural view. Scale bar = 10 μm.

**Fig. 10:**
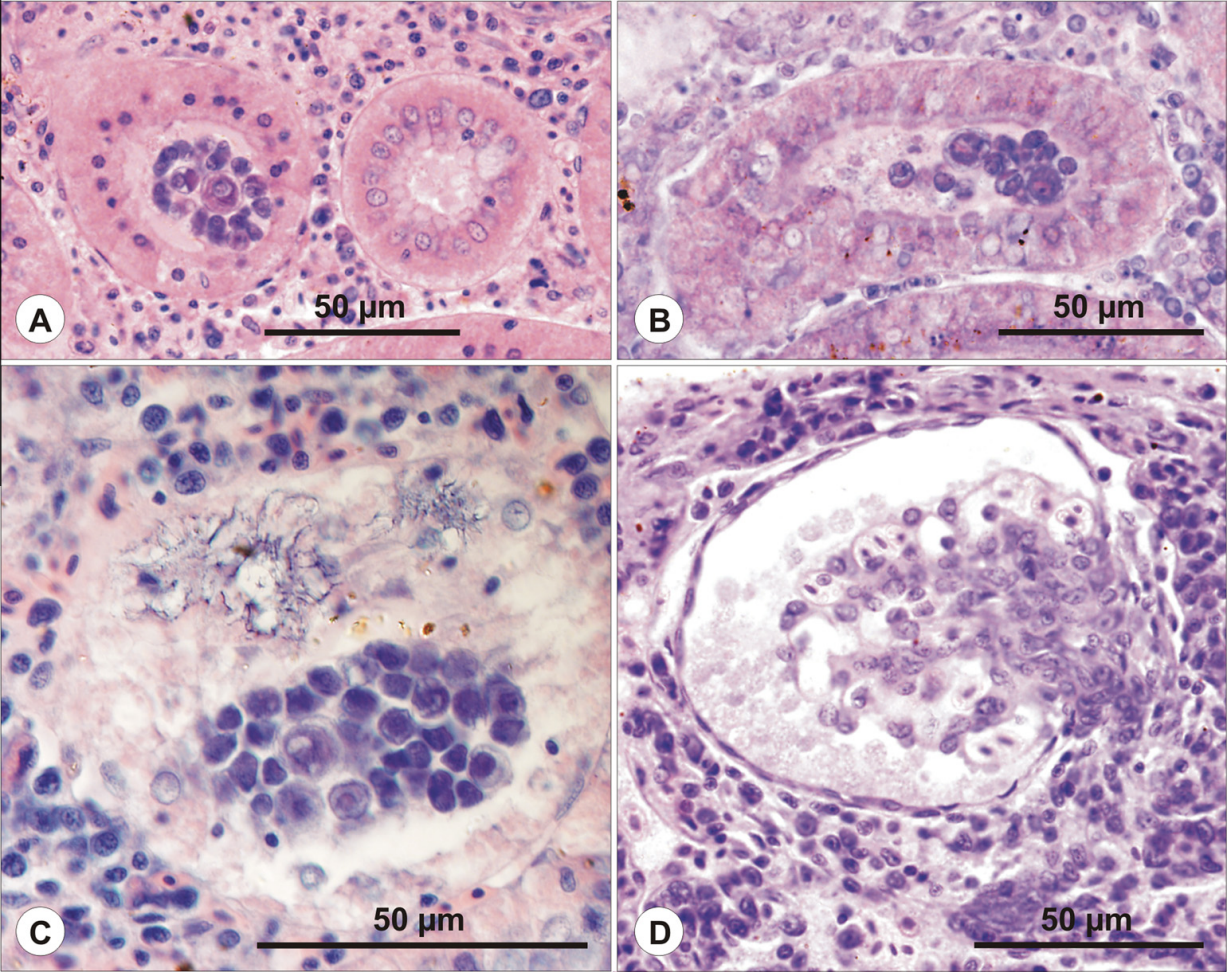
The histology of kidney infected with *Latyspora-*like organism. (A–B) Advanced stage of *Latyspora*-like organism infection in renal tubules of *Clupea harengus*. (A) Epithelium in infected segments of renal tubules consisting of cells with pyknotic nuclei suggestive of cellular necrosis. (B) Early stage of epithelial disintegration. (C) Loss of integrity of epithelium due to advanced necrotic changes. Basophilic remnants seen in necrotic epithelium indicate hypertrophy of some nuclei. (D) Hypertrophy of renal corpuscles containing foreign material in Bowman’s spaces was observed but cannot be solely associated with *Latyspora*-like organism infection.

***Latyspora-*like organism** (Fig. 1L–N, Fig. 9, Fig. 10).

Family Sinuolineidae Shulman, 1959.

Genus *Latyspora* Bartošová, Freeman, Yokoyama, Caffara and Fiala, 2010.

*Type host*: *C. harengus* Linnaeus, 1758, Atlantic herring; average standard length 20.9 cm.

*Other hosts*: unknown.

*Type locality*: Greenland Sea, part of the Billefjorden, Isfjorden, Petunia Bay in the central part of Svalbard archipelago (78° 69′ N, 16° 53′ E).

*Other localities:* none.

*Description of sporogonic stages*: disporic plasmodia globular in shape containing numerous refractile granules; plasmodia developing in renal tubules (attached to the epithelium and sometimes invading into epithelium); for dimensions see Table 1.

*Description of myxospores*: spores bean-shaped or trapezoidal from frontal view, oval from the apical view; both valves smooth with rounded shape; spore folds formed by the shell valve at its posterior pole; straight sutural line running perpendicularly between two spherical PCs of equal size, PCs located close together at anterior pole and oriented in the same direction, discharging sideways, PCs with a straight central shaft of the filament, 6–7 coils of polar filament; single sporoplasm with two nuclei; for dimensions see Table 1.

*Localization of sporogonic stages:* coelozoic; renal tubules.

*Prevalence*: 14% (9 of 66 kidney samples).

*Pathology*: advanced infection associated with alteration to the epithelium of renal tubules either by atrophy of epithelial cells and pyknosis of cell nuclei or complete loss of integrity of epithelium due to necrotic changes; hypertrophy of renal corpuscles caused by foreign material accumulated in dilated Bowman’s spaces not possible to unambiguously associate with infection (Fig. 10).

*Materials deposited*: DNA sample (nr. 1365) stored at −80 °C and paraffin blocks nrs. 695/09, 700/09, 704/09 stored in the Laboratory of Fish Protistology, Institute of Parasitology, BC ASCR; SSU (GenBank accession No. KF874234) and LSU (GenBank accession No. KF874225) rDNAs sequences.

*Remarks:* Classification of this species near the genus *Latyspora* is based on the current state of *Latyspora* taxonomy (Bartošová et al., 2011). The taxonomic status of *Latyspora-*like organism will be emended in the future when myxozoan taxonomy and in particular the genus *Latyspora* is revised. *Latyspora*-like organism differs morphologically from the genus *Latyspora* in one morphological characteristic: the sutural line is straight in *Latyspora*-like organism vs. sinuous in the type species *Latyspora scomberomori*. Other morphological and biological characteristics e.g. localization in the fish host fully correspond to *Latyspora* and phylogenetically, the genus type species *L. scomberomori* and *Latyspora*-like organism are distantly related.

### 3.3 Phylogenetic analyses

**Fig. 11:**
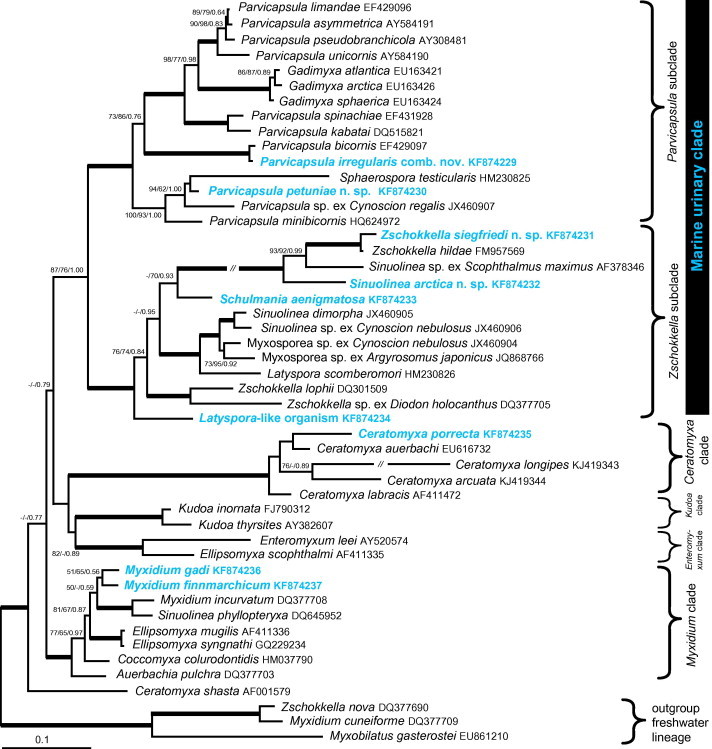
Maximum likelihood tree (-ln = 18360.2248) based on 52 SSU rDNA myxosporean sequences available in GenBank and newly obtained data (in bold blue colour) belonging to the marine urinary clade. Numbers at the nodes represent the bootstrap values and the Bayesian posterior probability (ML/MP/BI) gaining more than 50% support (ML and MP) and 0.5 posterior probability (BI), respectively. Bold branches lead to a node with a bootstrap support of ⩾95 and a Bayesian posterior probability of ⩾0.97. Scale bar is given under the tree. (For interpretation of the references to colour in this figure legend, the reader is referred to the web version of this article.)

Seven newly molecularly characterised myxosporeans clustered within the marine myxosporean lineage in the rDNA-based phylogenies (Fig. 11, Supplementary Fig. 1A). Phylogenetic tree based on five new LSU rDNA and concatenated analysis based on SSU + LSU rDNA shows the marine urinary clade monophyletic, however subclades are not well resolved (Supplementary Fig. 1A, B).

All species, except *Ceratomyxa porrecta*, clustered within the clade of marine myxosporeans mainly infecting the urinary bladder of fish i.e. the marine urinary clade according to Bartošová et al. (2011). *Ceratomyxa porrecta* branched in the *Ceratomyxa* clade (Supplementary Fig. 2B) with a close relationship to *C*. *auerbachi* described from the North Sea and Norwegian Sea region. SSU rDNA sequences of *M. gadi* and *M. finnmarchicum*, obtained in this study, clustered with sequences of these species currently available in GenBank (Supplementary Fig. 2A). Their sequence similarities within the species were higher than 98% (Supplementary Table 2). The analysis of the marine *Myxidium* clade confirmed the sister relationship of *M. gadi* and *M. bergense* and revealed the position of *M. finnmarchicum* as an early branching species closely related to *S. phyllopteryxa* and *M. incurvatum* (Supplementary Fig. 2A).

The marine urinary clade was enlarged by the addition of six newly sequenced species (Fig. 11). *Parvicapsula irregularis* was very closely related to pathogenic *P. bicornis* in both SSU and SSU + LSU rDNA-based trees with maximum bootstrap support (Fig. 11 and Supplementary Fig. 1A) and with a high sequence similarity of 98.1% (Supplementary Table 1). *Parvicapsula petuniae* clustered with *S. testicularis* with high nodal support in the SSU rDNA-based ML and BI (MP bootstrap support was low; Fig. 11) and their sequence similarity was 85.9% (Supplementary Table 1). Both newly obtained *Parvicapsula* sequences branched within the *ParvicapsuIa* subclade of the marine urinary clade with high nodal support in the SSU rDNA-based tree (Fig. 11). The *Zschokkella* subclade is enriched almost 1.5 times. *Latyspora-*like organism was the most basal species of the *Zschokkella* subclade and did not cluster with the type species *L. scomberomori* in any analyses (Fig. 11, Supplementary Fig. 1A). *Sinuolinea arctica* and *Zschokkella siegfriedi* clustered together with both *Z. hildae* and *Sinuolinea* sp. with high nodal support in the SSU and SSU + LSU rDNA-based phylogenies (Fig. 11 and Supplementary Fig. 1A). The clade of the four aforementioned species was characterised by the long branch in the phylogenetic trees (Fig. 11, Supplementary Fig. 1A). *Zschokkella siegfriedi* and *Z. hildae* were closely related (Fig. 11, Supplementary Fig. 1A) with relatively high sequence similarity of 97.2% (Supplementary Table 1). *Schulmania aenigmatosa* was revealed as the sister taxon to the abovementioned long-branching group with low nodal support in the SSU rDNA tree (Fig. 11). Concatenated analysis of SSU + LSU rDNA data supported the relationship of *S. aenigmatosa* with the group containing *L. scomberomori* and *S. dimorpha* (99% bootstrap support in ML) and revealed the long-branching group of two *Zschokkella* spp. and two *Sinuolinea* spp. inside the *Parvicapsula* subclade (Supplementary Fig. 1A). In addition, the topology within the marine urinary clade was identical after changing the outgroup (three *Ceratomyxa* species instead of three freshwater myxosporeans) in the analysis testing the influence of the selected outgroup on the resulting topology (tree not shown). Based on the three main alignments focused on the Myxosporea infecting urinary bladder, the marine urinary clade was a well resolved group with nodal supports (ML/MP/BI) of 87/76/1.00 in the SSU rDNA-based tree, 60/80/1.00 in the LSU rDNA-based tree and 100/99/0.92 in the SSU + LSU rDNA-based tree, respectively (Fig. 11, Supplementary Fig. 1A, B).

## 4 Discussion

All our findings of myxosporean species in this study are the most Northern records of the Myxosporea. *Myxidium gadi* has an extended geographic distribution around the north part of the Northern hemisphere. Polar cod, host of *Zschokkella siegfriedi*, is one of the most northerly distributed gadid fish, has a circumpolar distribution (Froese and Pauly, 2013). We can assume that most of the myxosporeans described from Svalbard have a wide distribution not limited by the Svalbard archipelago but not exceeding the range of their intermediate fish hosts.

Differences in prevalence and infection intensity of Myxosporea were detected in the fish hosts. The highest myxosporean prevalence and infection intensity was observed in benthopelagic fish *B. saida* infected with *Zschokkella siegfriedi* (43%) and in benthic fish *H. platessoides* infected with *P. irregularis* (44%). Generally, the prevalence of all four myxosporeans found in benthic fish *M. scorpius* was low, however, this host was infected with the highest number of myxosporean species (Table 1). The rich myxosporean fauna of *M*. *scorpius* was revealed during sequencing of selected microscopically myxosporean positive samples. Hidden mixed infections of *Myxidium finnmarchicum* and *M. gadi* revealed by PCR suggest the presence of presporogonic or sporogonic stages with low infection intensity, which can be easily overlooked or misidentified with stages of belonging to myxosporean species with high prevalence. The differences in infection intensity and parasite abundance between hosts may be explained by competition or other negative interactions among parasites in the fish host (Seppala et al., 2009).

Myxosporeans have not been reported on Svalbard or the surrounding marine environment so far except the finding of *Z. hildae* from *B. saida* (Køie, 2009). Therefore, we can only provide a comparison of myxosporean parasitofauna with geographic regions close to the Arctic. We chose the ratio of total number of myxosporean species found/number of dissected fish species as a measure to determine and compare the biodiversity among the regions. The ratio in our study (1.3) was very similar to the ratio (1.5) obtained in the study of gadid fish in the North Sea and Norwegian waters (Kalavati and MacKenzie, 1999). However, a much lower ratio (0.5) was recorded in 28 meso- and bathypelagic fish species from the continental shelf of Newfoundland and Labrador (water depth from 200 to 1000 m) (Khan et al., 1986). On the other side, one parasite per fish species in average (ratio 1.0) was revealed in mesopelagic fish in the North Atlantic (Yoshino and Noble, 1973). Therefore, it seems sea water depth rather than geographic distribution is an important factor influencing myxosporean fauna. Deep water fish (except benthopelagic) had the lowest ratio of myxosporeans per fish, which corresponds to observation of low parasite richness by Klimpel et al. (2006) in different meso- and bathypelagic fish. In contrast to high ratio of myxosporean infections observed in epi- and mesopelagic gadid fish (Kalavati and MacKenzie, 1999) which is one of the most dominant Arctic fish families. Although we did not dissect any Atlantic cod, whose parasitofauna has been well studied and includes a total 11 myxosporean species, we did examine fishes from the same depth range with similar parasite/host ratios. Heteroxenous parasite expansion is dependent also on the other host involved in the life cycle. It means that the myxosporean distribution area is restricted not only by fish abundance but also by the particular definitive host.

The morphologically simplified body organisation of the Myxozoa together with ancestral polymorphism and convergent evolution limit the number of characteristic features important for the classification of myxozoan genera (Avise, 2004). Moreover, myxosporeans often possess a certain degree of spore plasticity within evolutionary closely related species, especially within species clustering in the marine urinary clade (Fiala and Bartošová, 2010). Phylogenetic positions of myxosporeans obtained in this study strengthened the typical myxosporean discrepancies between taxonomy based on the morphological similarities and the observed phylogenetic relationships. This is evident in the close relationship of *P. petuniae* with *S. testicularis* and unrelated phylogenetic positions of the *Latyspora*-like organism and *L*. *scomberomori* as well as *Sinuolinea arctica* and *S*. *dimorpha*.

The myxospore shape of species from the marine urinary clade is very variable in comparison with shapes shared among species in other e.g. *Ceratomyxa* and *Kudoa* clades. Variability of the myxospore morphology can be seen in the position of PCs, twisting of the suture line around the valves and by alterations of the overall spore shape e.g. prolongation and broadening of the spore. Bartošová et al. (2011) investigated the evolution of the suture line in the marine urinary clade. They found the character of the suture line to be a typical homoplastic feature. Phylogenetic positions of the myxosporeans reported from Svalbard represented by the genera *Zschokkella*, *Parvicapsula*, *Sinuolinea*, *Latyspora* (all with curved or sinuous suture line) and *Schulmania* (straight suture line) supported the homoplasy of this feature. Moreover, Bartošová et al. (2011) traced the evolutionary character of the suture line i.e. sinuous or curved vs. straight on the SSU rDNA-based phylogeny. They found that an ancestor of the marine urinary clade possessed the curved suture line. *Latyspora-*like organism as the basal species of the *Zschokkella* subclade, has a remarkably straight suture line. Therefore, the evolutionary history of this feature would be different if we again trace this character on the tree which is in congruence with the statement of Bartošová et al. (2011) that poor taxon sampling influences the tracing of character evolution.

*Latyspora-*like organism is a problematic species, a taxonomic “hard nut to crack”, detailed in the description above. It has the straight suture line and differences in PC discharge and its phylogenetic position from the type species thus not allowing us to assign it to the genus *Latyspora*. The genera *Latyspora* and *Ceratomyxa* have very similar types of spores*,* nevertheless characters of suture line and position of PCs distinguish these two genera (Bartošová et al., 2011). The appropriate focus plane is crucial for the correct characterisation of the suture line as seen in the picture of *Latyspora*-like organism in Fig. 1N. We assume that the documentation of sinuous suture line of *L*. *scomberomori* (Bartošová et al., 2011) is questionable in that halo effect around the PCs, may have resulted in misinterpretation of the character of suture line. In any case, these two species are not phylogenetically closely related and thus *Latyspora*-like organism should not be assigned to the genus *Latyspora* which would make this genus polyphyletic. However, *Latyspora*-like organism may be representative of another so far undescribed genus.

Variability of the myxospore morphology was also studied at the level of a single species e.g. *Zschokkella pleomorpha* and *Bipteria formosa* during spore development. It was documented that the maturation process changes the shape and dimensions of the myxospore (Lom and Dyková, 1995) or formation of lateral wings (Karlsbakk and Køie, 2009). We assume that the lateral wings of *Schulmania aenigmatosa* undergo similar maturation changes as those in *B. formosa*. In these cases, it is important to provide morphometric data from the completely mature spores to avoid obtaining of misleading spore dimensions.

Speciation is not always accompanied by morphological change and many species remain undescribed (Bickford et al., 2007). Research on cryptic species has increased since molecular tools helped to distinguish closely related and morphologically similar or identical species. In our study, two species of the genus *Myxidium, M. finnmarchicum* and *M. gadi*, were hard to distinguish based on the morphology of the spores, which is a tool of classic myxosporean taxonomy. Both species occurred in the same host species and were present in low prevalence. The presence of these two different species was uncovered based on SSU rDNA screening of the sample and supported by a detailed morphometric analysis.

Another example is a cryptic myxosporean species found in Polar cod kidney tubules. Aseeva (2002) observed this myxosporean in Polar cod and classified it as *Zschokkella hildae* based on identical morphological and biological features*.* However, we revealed this myxosporean to be a cryptic species based on the genetic differences in the SSU rDNA and we named it as *Zschokkella siegfriedi. Zschokkella hildae* has been recorded in nine gadid fish including Arctic cod from Arctic region of Greenland (Køie et al., 2008b). Up to now SSU rDNA data of *Z. hildae* are available from the Atlantic cod only (Holzer et al., 2010). Hypothetically, more species can be revealed from the family Gadidae by molecular characterisation and they can represent hidden or misidentified species as in the case of *Z. siegfriedi* from Polar cod. The type host of *Z. hildae*, the Greater forkbeard *Phycis blennoides*, phylogenetically clusters apart from the other reported hosts of *Z. hildae* (Møller et al., 2002, Teletchea et al., 2006, Roa-Varon and Orti, 2009)*.* This may suggest that *Z*. *hildae* from the type host may not correspond to the myxosporean described (and sequenced) from Atlantic cod. More information about the *Zschokkella* subclade including increased taxon sampling effort together with providing biological characters from life cycles, development, ecology of definitive host etc. may lead to the radical taxonomic changes. Pleomorphic myxospores resembling *Zschokkella* morphotype and presence of the *Zschokkella* type species in the *Zschokkella* subclade may provoke assignment of all members of this subclade to the genus *Zschokkella*.

Discovery of *Z. siegfriedi*, morphologically identical species with *Z*. *hildae*, based on SSU rDNA sequence divergence underlines the importance of molecular data for species description and for parasite new host records. However, the level of myxosporean genetic interspecific dissimilarity is fluctuating, which do not allow simple use of arbitrary chosen level of genetic dissimilarity to discriminate between species. For example, members of the genus *Ceratomyxa* have much lower sequence difference up to 0.4% (Gunter and Adlard, 2009), which is in contrast to *Chloromyxum leydigi* with 1.8% intraspecific variation (Glesoon and Adlard, 2012). Similarly in this study, *Myxidium gadi* a generalist parasite of gadid fish has 1.2% of intraspecific variation and, on the other hand, sequence dissimilarity between *Parvicapsula limandae* and *P*. *assymetrica* is 0.9%, and among *Ellipsomyxa* spp. is even about 0.5%. As already discussed in Gunter and Adlard (2009), the level of DNA sequence difference must be assessed on a case to case basis using a whole evidence approach.

Marine myxosporean life cycles are poorly resolved with only few described ones for specifically *Ceratomyxa auerbachi*, *Gadimyxa atlantica*, *Sigmomyxa sphaerica*, two species of *Parvicapsula* and two species of *Ellipsomyxa*. All of them have a polychaete definitive host in their life cycle (Køie et al., 2004, Køie et al., 2007a, Køie et al., 2008a, Rangel et al., 2009, Karlsbakk and Køie, 2012, Køie et al., 2013). Lower levels of species richness may give polar regions an advantage for studying myxozoan life cycles compared to species-rich subtropical or tropical regions. Therefore, the Svalbard coast may be a suitable area for life cycle studies, supported by preliminary data on the life cycle of *Gadimyxa sphaerica* (results will be published elsewhere). We may hypothesise a polychaete worm as a host for *P. petuniae*, since the closely related *P. minibicornis* uses a freshwater polychaete, *Manayunkia speciosa* as a host (Bartholomew et al., 2006). Nevertheless, the elucidation of the life cycles of myxosporeans from Svalbard region is a task for future studies.

Except of the universal SSU rDNA marker, we also sequenced LSU rDNA of Myxosporea in order to add more molecular data to our analyses. Nevertheless, the single LSU rDNA analysis of the marine urinary clade contained significantly less taxa compared with the SSU rDNA analysis. This discrepancy in amount of the characters for particular taxa may cause the different topological pattern of SSU vs SSU + LSU rDNA analyses. Moreover, LSU rDNA has higher phylogenetic signal and may suppress the signal of SSU rDNA leading to different topology (Bartošová et al., 2009).

Our research indicates that increased taxon sampling effort is needed to elucidate myxosporean relationships, mainly of species from the urinary system clustering in the marine urinary clade. This clade accommodates many diverse myxosporean morphotypes and therefore, new molecular data for species from urinary systems of marine fish, especially those classified to genera with missing molecular data, are needed. There is also an obvious importance of studying parasites from the Arctic as a region most influenced by climate change (Post et al., 2009) in order to monitor its changing parasitofauna. New phylogenetic data from species infecting urinary tract contribute to the knowledge of evolution of the marine myxosporeans.

## 5 Conclusions

Our focus on myxosporeans of benthic and pelagic fish collected in the central part of Svalbard revealed the presence of several new myxosporean species. Results of the present study increase the species richness of myxosporeans in a polar region as well broaden the spectrum of their hosts and their distribution in the studied area. We mostly found myxosporean species infecting the urinary tract that are distinguished by the morphologically variable spores and classified to five myxosporean genera. These species clustered together based on shared tissue tropism rather than their myxospore morphology. Based on adequate taxon sampling and SSU and LSU rDNA-based phylogeny, we discussed evolutionary trends within the marine urinary clade.
