# Supplementary figures and images for: Myxozoa in high Arctic: Survey on the central part of Svalbard archipelago
Source: Int J Parasitol Parasites Wildl. 2014 Feb 26;3(1):41–56. doi: 10.1016/j.ijppaw.2014.02.001 (PMC4047956; doi:10.1016/j.ijppaw.2014.02.001)

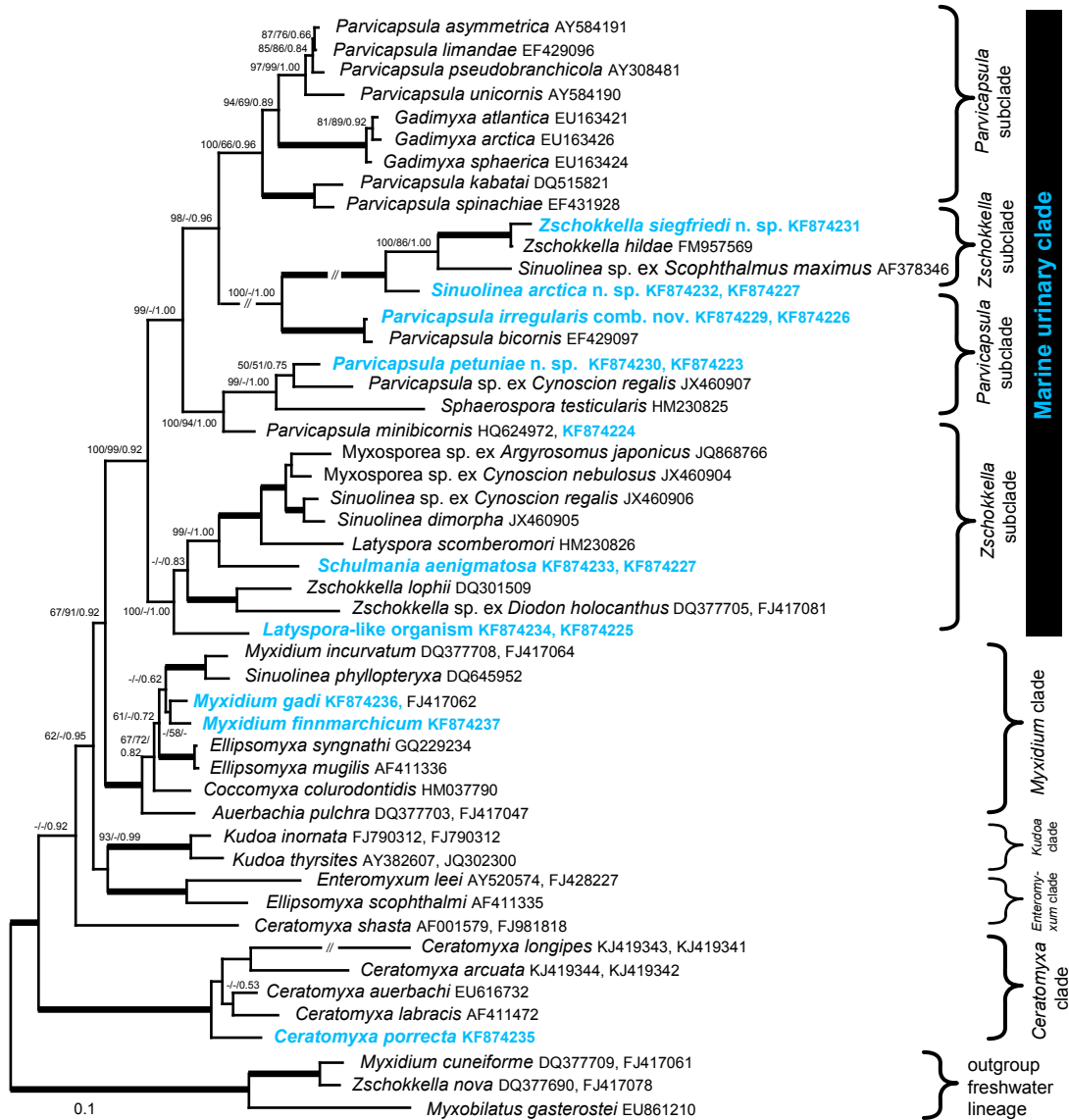

A.

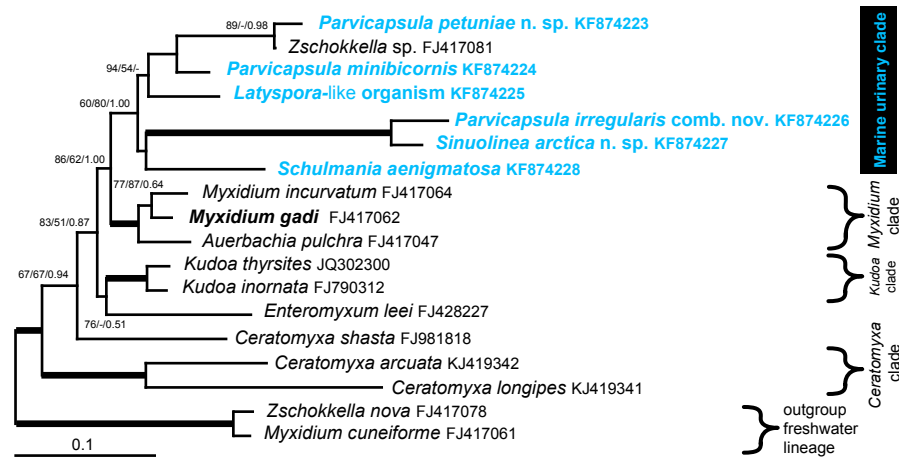

B.

Supplement: Supplementary Fig. 1 [file mmc1.pdf]

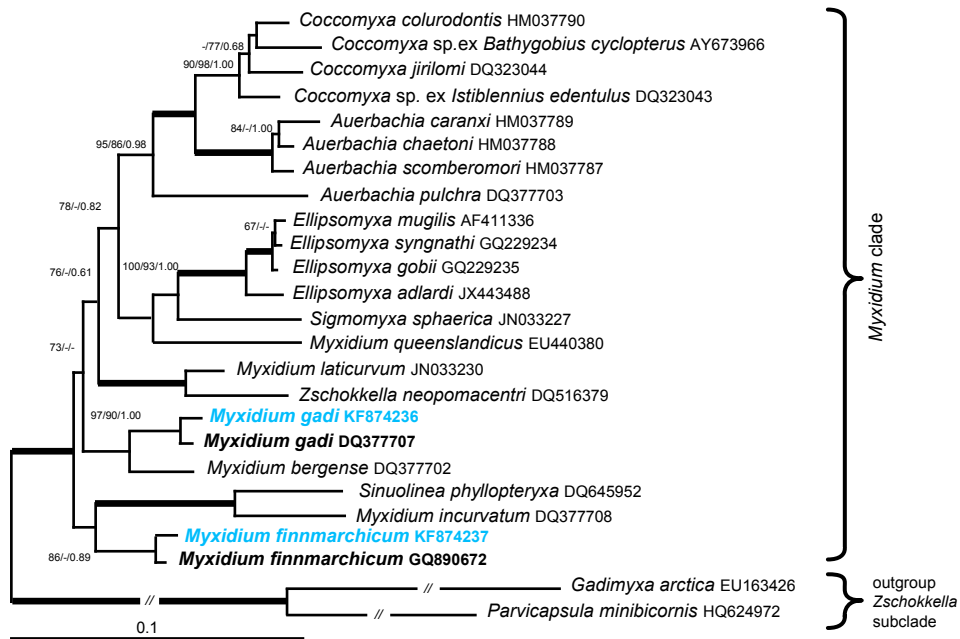

A.

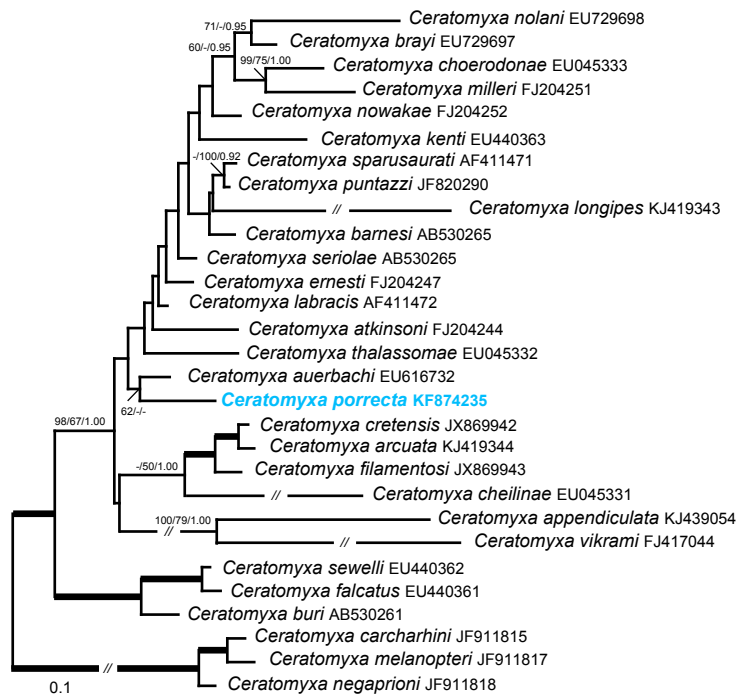

B.

Supplement: Supplementary Fig. 2 [file mmc2.pdf]
