# Supplementary material for: Myxozoa in high Arctic: Survey on the central part of Svalbard archipelago
Source: Int J Parasitol Parasites Wildl. 2014 Feb 26;3(1):41–56. doi: 10.1016/j.ijppaw.2014.02.001 (PMC4047956; doi:10.1016/j.ijppaw.2014.02.001)
Supplement: Supplementary Table 1 [file mmc3.docx]

|  | **Species from the Marine urinary clade** | **1** | **2** | **3** | **4** | **5** | **6** | **7** | **8** | **9** | **10** | **11** | **12** | **13** | **14** | **15** | **16** | **17** | **18** | **19** | **20** | **21** | **22** | **23** | **24** | **25** | **26** | 27 |
| --- | --- | --- | --- | --- | --- | --- | --- | --- | --- | --- | --- | --- | --- | --- | --- | --- | --- | --- | --- | --- | --- | --- | --- | --- | --- | --- | --- | --- |
| **1** | *Zschokkella siegfriedi* |  |  |  |  |  |  |  |  |  |  |  |  |  |  |  |  |  |  |  |  |  |  |  |  |  |  |  |
| **2** | *Zschokkella hildae* | 97.2 |  |  |  |  |  |  |  |  |  |  |  |  |  |  |  |  |  |  |  |  |  |  |  |  |  |  |
| **3** | *Sinuolinea* sp. ex *Scophthalmus maximus* | 84.9 | 82.2 |  |  |  |  |  |  |  |  |  |  |  |  |  |  |  |  |  |  |  |  |  |  |  |  |  |
| **4** | *Sinuolinea arctica* | 83.5 | 81.1 | 81.8 |  |  |  |  |  |  |  |  |  |  |  |  |  |  |  |  |  |  |  |  |  |  |  |  |
| **5** | *Schulmania aenigmatosa* | 75.4 | 73.6 | 70.8 | 74.4 |  |  |  |  |  |  |  |  |  |  |  |  |  |  |  |  |  |  |  |  |  |  |  |
| **6** | *Sinuolinea dimorpha* | 72.4 | 69.6 | 69.8 | 70.9 | 82.5 |  |  |  |  |  |  |  |  |  |  |  |  |  |  |  |  |  |  |  |  |  |  |
| **7** | *Sinuolinea* sp. ex *Cynoscion nebulosus* | 73.2 | 70.2 | 69.9 | 71.5 | 82.5 | 92.2 |  |  |  |  |  |  |  |  |  |  |  |  |  |  |  |  |  |  |  |  |  |
| **8** | Myxosporea sp. ex *Cynoscion nebulosus* | 71.7 | 70.6 | 69.5 | 70.5 | 81.5 | 89.8 | 89.8 |  |  |  |  |  |  |  |  |  |  |  |  |  |  |  |  |  |  |  |  |
| **9** | Myxosporea sp. *Argyrosomus japonicus* | 72.3 | 68.9 | 64.8 | 68.8 | 79.7 | 89.5 | 89.7 | 90.3 |  |  |  |  |  |  |  |  |  |  |  |  |  |  |  |  |  |  |  |
| **10** | *Latyspora scomberomori* | 72.3 | 69.8 | 70.9 | 72.7 | 80.7 | 83.1 | 83.6 | 83.2 | 82.6 |  |  |  |  |  |  |  |  |  |  |  |  |  |  |  |  |  |  |
| **11** | *Zschokkella lophii* | 75.2 | 74.1 | 73.3 | 75.9 | 85.6 | 84.2 | 84.1 | 85.8 | 83.7 | 82.4 |  |  |  |  |  |  |  |  |  |  |  |  |  |  |  |  |  |
| **12** | *Zschokkella* sp. ex *Diodon holocanthus* | 70.9 | 68.5 | 68.7 | 71.2 | 79.1 | 77.5 | 78.2 | 78.2 | 77.6 | 76.0 | 84.9 |  |  |  |  |  |  |  |  |  |  |  |  |  |  |  |  |
| **13** | *Latyspora-*like organism | 76.4 | 74.2 | 72.8 | 76.3 | 86.1 | 83.6 | 84.1 | 85.3 | 82.5 | 83.6 | 84.8 | 81.6 |  |  |  |  |  |  |  |  |  |  |  |  |  |  |  |
| **14** | *Parvicapsula limandae* | 76.0 | 69.4 | 69.7 | 69.4 | 78.2 | 77.4 | 78.1 | 77.6 | 72.7 | 76.7 | 79.8 | 76.5 | 82.4 |  |  |  |  |  |  |  |  |  |  |  |  |  |  |
| **15** | *Parvicapsula asymmetrica* | 75.4 | 69.5 | 69.2 | 70.9 | 79.4 | 76.5 | 76.9 | 79.0 | 76.3 | 76.0 | 78.3 | 75.6 | 80.1 | 99.1 |  |  |  |  |  |  |  |  |  |  |  |  |  |
| **16** | *Parvicapsula pseudobranchicola* | 75.9 | 70.3 | 68.9 | 71.0 | 79.3 | 76.1 | 76.0 | 79.3 | 76.0 | 75.4 | 77.7 | 75.5 | 79.7 | 94.5 | 94.5 |  |  |  |  |  |  |  |  |  |  |  |  |
| **17** | *Parvicapsula unicornis* | 74.4 | 69.6 | 69.1 | 71.8 | 78.9 | 75.9 | 76.3 | 78.7 | 75.5 | 75.2 | 76.0 | 74.8 | 79.7 | 90.5 | 91.1 | 90.4 |  |  |  |  |  |  |  |  |  |  |  |
| **18** | *Gadimyxa atlantica* | 74.4 | 72.1 | 72.7 | 73.8 | 81.6 | 80.6 | 80.5 | 79.4 | 79.4 | 79.4 | 79.9 | 78.2 | 81.7 | 86.6 | 87.1 | 87.2 | 87.0 |  |  |  |  |  |  |  |  |  |  |
| **19** | *Gadimyxa arctica* | 74.2 | 72.1 | 72.9 | 74.1 | 81.8 | 80.8 | 80.5 | 79.7 | 79.4 | 79.5 | 80.3 | 78.4 | 82.3 | 86.7 | 87.3 | 87.3 | 87.5 | 98.8 |  |  |  |  |  |  |  |  |  |
| **20** | *Gadimyxa sphaerica* | 74.3 | 72.4 | 72.6 | 73.7 | 82.2 | 80.6 | 80.2 | 79.6 | 78.7 | 79.5 | 80.2 | 77.7 | 82.0 | 86.7 | 87.3 | 87.1 | 87.0 | 98.3 | 97.9 |  |  |  |  |  |  |  |  |
| **21** | *Parvicapsula spinachiae* | 75.7 | 69.5 | 69.3 | 70.6 | 79.0 | 79.4 | 79.7 | 79.6 | 74.9 | 77.5 | 80.4 | 77.6 | 82.7 | 84.3 | 84.7 | 85.0 | 86.6 | 84.1 | 84.1 | 84.0 |  |  |  |  |  |  |  |
| **22** | *Parvicapsula kabatai* | 75.1 | 69.9 | 68.1 | 69.6 | 77.6 | 78.2 | 78.8 | 78.5 | 74.9 | 77.3 | 80.3 | 78.0 | 81.9 | 82.9 | 84.7 | 85.0 | 85.8 | 83.1 | 83.2 | 82.3 | 88.0 |  |  |  |  |  |  |
| **23** | *Parvicapsula bicornis* | 73.5 | 69.7 | 67.7 | 69.7 | 78.0 | 77.5 | 78.3 | 78.3 | 74.8 | 75.6 | 78.0 | 76.7 | 80.5 | 82.0 | 82.8 | 83.0 | 82.0 | 80.9 | 80.5 | 80.4 | 82.3 | 82.2 |  |  |  |  |  |
| **24** | *Parvicapsula irregularis* | 73.8 | 69.7 | 68.4 | 71.9 | 78.9 | 77.4 | 78.0 | 79.3 | 76.6 | 75.4 | 77.8 | 76.3 | 78.9 | 82.7 | 81.4 | 81.0 | 80.7 | 82.0 | 81.8 | 81.5 | 82.6 | 83.0 | 98.1 |  |  |  |  |
| **25** | *Sphaerospora testicularis* | 73.2 | 69.9 | 68.4 | 71.0 | 79.1 | 76.7 | 77.4 | 78.1 | 75.4 | 75.4 | 77.3 | 75.4 | 78.4 | 81.9 | 81.2 | 80.4 | 80.4 | 82.2 | 82.2 | 82.4 | 82.1 | 81.9 | 80.8 | 80.1 |  |  |  |
| **26** | *Parvicapsula petuniae* | 70.7 | 67.8 | 65.4 | 67.1 | 74.8 | 74.5 | 74.8 | 74.5 | 72.4 | 73.7 | 79.5 | 74.1 | 81.9 | 81.8 | 83.3 | 83.6 | 83.3 | 81.3 | 81.0 | 81.0 | 81.1 | 81.1 | 82.5 | 84.2 | 85.9 |  |  |
| **27** | *Parvicapsula* sp. ex *Cynoscion regalis* | 72.6 | 66.2 | 57.8 | 64.8 | 71.2 | 72.0 | 71.5 | 71.8 | 69.2 | 71.6 | 78.4 | 75.8 | 78.5 | 75.3 | 80.7 | 81.2 | 80.8 | 77.6 | 78.1 | 77.3 | 72.6 | 73.6 | 77.1 | 79.4 | 78.7 | 83.8 |  |
| **28** | *Parvicapsula minibicornis* | 72.4 | 66.7 | 67.4 | 69.5 | 75.8 | 77.1 | 77.1 | 77.8 | 75.6 | 75.0 | 78.7 | 74.6 | 80.1 | 82.0 | 80.4 | 79.6 | 81.2 | 83.2 | 83.4 | 82.9 | 82.9 | 83.0 | 82.1 | 81.7 | 83.6 | 85.3 | 80.6 |
