# Supplementary material for: Myxozoa in high Arctic: Survey on the central part of Svalbard archipelago
Source: Int J Parasitol Parasites Wildl. 2014 Feb 26;3(1):41–56. doi: 10.1016/j.ijppaw.2014.02.001 (PMC4047956; doi:10.1016/j.ijppaw.2014.02.001)
Supplement: Supplementary Table 2 [file mmc4.docx]

|  | **Species from the *Myxidium* clade** | **1** | **2** | **3** | **4** | **5** | **6** | **7** | **8** | **9** | **10** | **11** | **12** | **13** | **14** | **15** | **16** | **17** | **18** | **19** | **20** | **21** | **22** | **23** | **24** |
| --- | --- | --- | --- | --- | --- | --- | --- | --- | --- | --- | --- | --- | --- | --- | --- | --- | --- | --- | --- | --- | --- | --- | --- | --- | --- |
| **1** | *Myxidium gadi* |  |  |  |  |  |  |  |  |  |  |  |  |  |  |  |  |  |  |  |  |  |  |  |  |
| **2** | *Myxidium gadi* ex *Myoxocephalus scorpius* | 98.8 |  |  |  |  |  |  |  |  |  |  |  |  |  |  |  |  |  |  |  |  |  |  |  |
| **3** | *Myxidium finnmarchicum* | 95.6 | 94.1 |  |  |  |  |  |  |  |  |  |  |  |  |  |  |  |  |  |  |  |  |  |  |
| **4** | *Myxidium finnmarchicum* ex *Myoxocephalus scorpius* | 95.5 | 94.4 | 98.4 |  |  |  |  |  |  |  |  |  |  |  |  |  |  |  |  |  |  |  |  |  |
| **5** | *Myxidium bergense* | 96.8 | 95.0 | 93.3 | 92.2 |  |  |  |  |  |  |  |  |  |  |  |  |  |  |  |  |  |  |  |  |
| **6** | *Myxidium laticurvum* | 94.6 | 91.6 | 92.3 | 91.2 | 92.7 |  |  |  |  |  |  |  |  |  |  |  |  |  |  |  |  |  |  |  |
| **7** | *Myxidium queenslandicus* | 93.0 | 92.4 | 91.6 | 91.3 | 92.6 | 93.1 |  |  |  |  |  |  |  |  |  |  |  |  |  |  |  |  |  |  |
| **8** | *Mixidium incurvatum* | 91.2 | 90.4 | 91.3 | 90.2 | 90.7 | 90.3 | 90.3 |  |  |  |  |  |  |  |  |  |  |  |  |  |  |  |  |  |
| **9** | *Sinuolinea phyllopteryxa* | 91.2 | 89.2 | 90.5 | 89.1 | 90.6 | 90.1 | 90.5 | 93.8 |  |  |  |  |  |  |  |  |  |  |  |  |  |  |  |  |
| **10** | *Auerbachia pulchra* | 93.3 | 90.3 | 91.5 | 90.0 | 92.1 | 91.4 | 91.8 | 89.3 | 88.6 |  |  |  |  |  |  |  |  |  |  |  |  |  |  |  |
| **11** | *Auerbachia caranxi* | 93.2 | 93.3 | 93.1 | 92.0 | 93.0 | 92.7 | 92.7 | 90.3 | 90.9 | 92.9 |  |  |  |  |  |  |  |  |  |  |  |  |  |  |
| **12** | *Auerbachia scomberoidi* | 93.8 | 94.0 | 93.1 | 92.0 | 93.3 | 92.8 | 93.3 | 90.6 | 91.1 | 93.2 | 98.0 |  |  |  |  |  |  |  |  |  |  |  |  |  |
| **13** | *Auerbachia chaetodoni* | 94.2 | 94.2 | 93.6 | 92.6 | 93.4 | 93.0 | 92.9 | 90.7 | 91.5 | 93.5 | 98.2 | 98.8 |  |  |  |  |  |  |  |  |  |  |  |  |
| **14** | *Coccomyxa colurodontidis* | 93.8 | 93.5 | 93.4 | 92.4 | 93.4 | 93.0 | 92.9 | 90.5 | 91.0 | 93.1 | 95.4 | 96.0 | 96.0 |  |  |  |  |  |  |  |  |  |  |  |
| **15** | *Ellipsomyxa syngnathi* | 93.9 | 91.3 | 91.4 | 89.9 | 91.6 | 91.5 | 93.5 | 89.5 | 90.5 | 91.3 | 93.0 | 93.5 | 93.8 | 93.2 |  |  |  |  |  |  |  |  |  |  |
| **16** | *Ellipsomyxa gobii* | 93.9 | 91.2 | 91.3 | 89.7 | 91.6 | 91.5 | 93.6 | 89.6 | 90.4 | 91.3 | 92.8 | 93.2 | 93.5 | 93.1 | 99.5 |  |  |  |  |  |  |  |  |  |
| **17** | *Ellipsomyxa mugilis* | 94.5 | 91.6 | 91.4 | 90.0 | 91.9 | 92.0 | 93.5 | 90.1 | 90.5 | 91.7 | 93.0 | 93.4 | 93.9 | 93.6 | 99.6 | 99.5 |  |  |  |  |  |  |  |  |
| **18** | *Ellipsomyxa adlardi* | 93.7 | 91.5 | 91.7 | 90.6 | 91.9 | 91.3 | 93.6 | 89.9 | 90.2 | 90.9 | 93.2 | 93.8 | 93.9 | 93.1 | 97.4 | 97.5 | 97.8 |  |  |  |  |  |  |  |
| **19** | *Zschokkella neopomacentri* | 94.1 | 90.4 | 90.5 | 89.3 | 91.8 | 96.0 | 91.8 | 90.2 | 90.0 | 91.1 | 92.1 | 92.4 | 92.5 | 92.4 | 91.5 | 91.4 | 91.5 | 91.5 |  |  |  |  |  |  |
| **20** | *Coccomya jirilomi* | 93.6 | 90.5 | 91.3 | 89.6 | 92.3 | 91.4 | 92.3 | 89.8 | 89.6 | 92.2 | 95.6 | 95.9 | 96.0 | 97.6 | 91.8 | 91.7 | 91.8 | 92.1 | 91.0 |  |  |  |  |  |
| **21** | *Coccomyxa* sp. ex *Bathygobius cyclopterus* | 93.8 | 91.0 | 91.2 | 90.2 | 91.7 | 91.6 | 92.0 | 89.3 | 89.8 | 91.8 | 95.8 | 95.8 | 96.4 | 97.6 | 92.1 | 92.0 | 92.0 | 91.9 | 91.1 | 96.1 |  |  |  |  |
| **22** | *Coccomyxa* sp. ex *Istiblennius edentulus* | 93.7 | 91.9 | 91.8 | 90.9 | 92.6 | 92.3 | 92.2 | 89.9 | 90.2 | 92.3 | 95.6 | 95.6 | 96.0 | 97.1 | 92.3 | 92.3 | 92.2 | 92.3 | 91.3 | 96.7 | 96.6 |  |  |  |
| **23** | *Sigmomyxa sphaerica* | 93.0 | 90.5 | 91.0 | 90.2 | 90.6 | 91.7 | 93.5 | 89.4 | 89.0 | 91.4 | 92.3 | 92.5 | 92.8 | 92.5 | 93.4 | 93.3 | 93.6 | 93.4 | 91.8 | 91.1 | 90.7 | 91.3 |  |  |
| **24** | *Gadimyxa arctica* | 83.4 | 82.5 | 82.4 | 81.7 | 82.3 | 82.5 | 82.0 | 81.4 | 80.9 | 83.5 | 81.7 | 82.0 | 82.2 | 82.4 | 82.7 | 82.7 | 82.6 | 82.4 | 81.9 | 81.7 | 81.8 | 82.4 | 83.4 |  |
| **25** | *Parvicapsula minibicornis* | 84.5 | 82.8 | 83.1 | 81.7 | 83.0 | 82.3 | 81.5 | 82.0 | 82.4 | 82.8 | 84.0 | 84.0 | 83.9 | 83.8 | 82.1 | 82.1 | 83.7 | 82.6 | 82.5 | 83.5 | 83.7 | 84.3 | 82.0 | 83.4 |
